# TGF-β1 regulates chondrocyte proliferation and extracellular matrix synthesis via circPhf21a-Vegfa axis in osteoarthritis

**DOI:** 10.1186/s12964-022-00881-9

**Published:** 2022-05-30

**Authors:** Shiyuan Lin, Huizi Li, Biao Wu, Jie Shang, Ning Jiang, Rong Peng, Baizhou Xing, Xianghe Xu, Huading Lu

**Affiliations:** 1 Department of Orthopaedics, The Fifth Affiliated Hospital of Sun Yat-Sen University, No. 52, Meihua East Road, Xiangzhou District, Zhuhai, 519000 Guangdong China; 2 Department of General Surgery, The Second Affiliated Hospital of Nanchang University, Nanchang, 330000 Jiangxi Province China

**Keywords:** Chondrocyte, circRNA, Osteoarthritis, TGF-β1, Vegfa

## Abstract

**Background:**

The transforming growth factor-beta (TGF-β) signaling pathway is an important pathway associated with the pathogenesis of osteoarthritis (OA). This study was to investigate the involvement of circRNAs in the TGF-β signaling pathway.

**Methods:**

Cell Counting Kit-8 (CCK-8) assay and 5-ethynyl-2′-deoxyuridine (EdU) assay were used to detect the proliferation of primary mouse chondrocytes (PMCs). RNA-sequencing together with bioinformatics analysis were used to systematically clarify TGF-β1 induced alternations of circRNAs in PMCs. The regulatory and functional role of circPhf21a was examined in PMCs. Downstream targets of circPhf21a were explored by RNA-sequencing after overexpression of circPhf21a and verified by RT-qPCR in PMCs. Finally, the role and mechanism of circPhf21a in OA were explored in mouse models.

**Results:**

We found that TGF-β1 promoted the proliferation of PMCs. Meanwhile, RT-qPCR and western blotting indicated that TGF-β1 promoted extracellular matrix (ECM) anabolism. RNA-sequencing revealed that a total of 36 circRNAs were differentially expressed between PMCs treated with and without TGF-β1. Of these, circPhf21a was significantly decreased by TGF-β1. Furthermore, circPhf21a knockdown promoted the proliferation and ECM synthesis of PMCs, whereas overexpression of circPhf21a showed the opposite effects. Mechanically, the expression profiles of the mRNAs revealed that Vegfa may be the target of circPhf21a. Additionally, we found that circPhf21a was significantly upregulated in the mouse OA model, and inhibition of circPhf21a significantly relieved the progression of OA.

**Conclusions:**

Our results found that TGF-β1 promoted the proliferation and ECM synthesis of PMCs via the circPhf21a-Vegfa axis, which may provide novel therapeutic targets for OA treatment.

**Video abstract**

**Supplementary Information:**

The online version contains supplementary material available at 10.1186/s12964-022-00881-9.

## Background

Osteoarthritis (OA) is one of the most frequent degenerative diseases of the joints, which is characterized by cartilage destruction and bony overgrowth in the form of osteophytes and subchondral thickening [1]. OA seriously threatens the quality of life of affected patients and causes huge social and medical burdens globally [2]. Owing to the aging population and epidemic of metabolic syndrome, the prevalence of OA is increasing rapidly in recent years [3, 4]. The prevalence of knee OA had 2.1-fold higher in the modern postindustrial era than that in the early industrial era in the United States, while the prevalence of OA showed an approximate 1.2% increase per year in the UK [5, 6].

The key mechanisms triggering the progression of OA were largely unclear, but accumulating evidence indicated that the TGF-β1 signaling pathway played a critical role in the pathogenesis of OA through participating in regulating cartilage damage, osteophyte formation and synovial fibrosis [7–9]. TGF-βs binding to the corresponding receptors induced the phosphorylation and activation of Smad signaling pathways, which in turn gave rise to differential expression of several TGF-β1 related genes in OA [10, 11]. Many studies revealed that suppression of TGF-β1 activity effectively inhibited the progression of OA in vitro and vivo, which suggested the therapeutic potentials of TGF-β1 in OA treatment [8, 12, 13]. However, repression of TGF-β1 maybe not be a promising therapeutic option for OA when considering the crucial roles of TGF-β1 in cartilage homeostasis and repair [14, 15]. Additionally, increasing studies indicated TGF-β1 was also a chondroprotective factor in cartilage maintenance and TGF-β1 released by mesenchymal stromal cells attenuated the development of osteoarthritis [16, 17]. Accordingly, the dual roles of the TGF-β1 during osteoarthritis development might also hinder the routine clinical application of drugs targeting TGF-β1 in OA patients at different stages [18]. Therefore, to turn to identify TGF-β1 related genes participating in OA progression may provide novel therapeutic targets for OA.

Circular RNAs (circRNAs), a type of RNA molecule with a covalently closed continuous loop, extensively existed in eukaryotes with tissue-specific and cell-specific patterns [19]. Some researchers showed that circRNAs may act as the central regulators in some biological processes [20]. Also, increasing studies demonstrated that circRNAs were associated with the initiation and development of several diseases including OA [21–23]. Zhou et al. found that a total of 255 circRNAs were differentially-expressed in IL-1β-treated mouse articular chondrocytes [24]. Furthermore, they identified that circRNA_Atp9b participated in OA progression through sponging miR-138-5p in chondrocytes [25]. Shen and coworkers showed that circSERPINE2 overexpression alleviated the progression of OA via miR-1271/ERG pathway [26]. Interestingly, previous studies revealed that several circRNAs were also involved in the TGF-β1 signaling pathway [27, 28], but the potential roles of TGF-β1 related circRNAs in OA were largely unclear. Therefore, it was essential to systematically identify the expression profiles of TGF-β1 related circRNAs in OA.

In the current study, we found that TGF-β1 promoted the proliferation and extracellular matrix (ECM) synthesis of primary mouse chondrocytes (PMCs). In addition, we identified a circRNA (termed circPhf21a) in TGF-β1 treated PMCs, and systemically investigated its role in vitro and animal models of OA.

## Materials and methods

### Isolation and culture of PMCs

PMCs were isolated from the knee cartilage of neonatal C57BL/6 mice within 5–6 days after birth [29]. In brief, the knee articular cartilage tissues were collected and cut into small pieces. Then, the samples were digested with 0.25% collagenase (Roche, cat. no. 11088882001) in a shaking incubator at 37 °C for 6 h. The pellets containing chondrocytes were filtered through a 70 μm cell strainer and collected by centrifugation at 1000 g for 5 min at room temperature. The harvested chondrocytes were cultured in Dulbecco’s Modified Eagle Medium/Nutrient Mixture F-12 (Gibco, DMEM/F-12, cat. no. C11330500BT) accompanied with 10% fetal bovine serum (Gibco, cat. no. 10099141) and 1% penicillin/streptomycin (Sigma, cat. no. P0781) at 37 °C with 5% CO2 and 95% air. PMCs within the fourth passage were used for the current experiments. This study was approved by the Institutional Animal Care and Use Committee (IACUC) of the Fifth Affiliated Hospital of Sun Yat-Sen University.

### Cell Counting Kit-8 (CCK-8) assay

PMCs were suspended and seeded into 96-well plates at a density of 2 × 10^3^ cells/well. Each well was filled with 100 μL culture medium. After incubated with 0, 1 or 5 ng/ml TGF-β1 for 0 h, 24 h, 48 h or 72 h, a total of 10 μL CCK-8 (Japan, Dojindo, cat. no. CK04) was added to each well. The cells were incubated in the solution for 4 h. The absorbance values were measured at 450 nm using a luminometer (BioTek uQuant, USA).

### EdU assay

To analyze PCMs proliferation, we performed EdU staining according to the manufacturer’s instructions using the BeyoClick^™^ EdU Cell Proliferation Kit with Alexa Fluor 555 (Beyotime Biotechnology, cat. no. C0075S). Briefly, the cells were incubated for 2 h at 37 °C after EdU was added to the culture media at a final concentration of 10 μM. After labeling, the cells were fixed for 15 min with 4% PFA and permeabilized for 10 min with 0.2% Triton X-100. The cells were then stained in the dark for 30 min with click reaction solution before being counterstained with Hoechst 33342. Under fluorescence microscopy (Olympus, Tokyo, Japan), EdU positive cells were counted and the percentage of EdU positive cells was reported.

### RNA extraction and RT-qPCR

RNA isolation of mouse articular cartilage and PMCs were performed according to our previous methods [30]. cDNA was synthesized using RevertAid First Strand cDNA Synthesis Kit (Thermo Fisher, cat. no. K1622) according to the manufacturer’s instructions. CircRNA was amplified by divergent primers. The PCR was performed in CFX96^™^ Real-Time System (Bio-Rad, USA). RT-qPCR reaction was performed using Forget-Me-Not^™^ EvaGreen^®^ qPCR Master Mix (Biotium, cat. no. 31042-1) in CFX96^™^ Real-Time PCR Detection Systems (Bio-Rad, USA). The expression level was determined by the threshold cycle (Ct), and relative expression levels were calculated using the 2^−∆∆CT^ method with Gapdh as the reference gene. The primers utilized in the current investigation were shown in Additional file 1: Table S1. Col2a1 and Aggrecan are major components of the extracellular matrix, while Mmp13, Adamts4 and Mmp3 are the main enzymes that degrade the extracellular matrix [26, 31–33]. So, we chose these genes for analysis in PMCs.

### Western blotting

The total protein of articular cartilage or PMCs was extracted by RIPA lysis buffer (Solarbio Biotech, cat. no. R0010) containing 1% PMSF protease inhibitor (Solarbio Biotech, cat. no. P0100). The supernatants were collected by centrifugation at 14,000 g for 15 min at 4 °C. The protein concentrations were measured by Enhanced Bicinchoninic Acid (BCA) protein assay kit (Beyotime Biotechnology, cat. no. P0010). The total protein was separated using 10% SDS-PAGE gels (EpiZyme, cat. no. PG112) and a Prestained Protein Ladder (Thermo Scientific, cat. no. 26616) was used as a marker. Then the separated protein was transferred onto polyvinylidene difluoride (PVDF) membranes (Bio-Rad, cat. no. 1620177). 5% skimmed milk was used to block the PVDF membrane at room temperature for 1 h. The membrane was then incubated with primary anti-Mmp13 (diluted 1:1000, Proteintech, cat. no. 18165-1-AP), anti-Col2a1 (diluted 1:1000, ABclonal, cat. no. A1560), anti-Vegfa (diluted 1:1000, ABclonal, cat. no. A12303) and anti-Gapdh (diluted 1:8000; Proteintech, cat. no. 60004-1-Ig) overnight at 4 °C. After washing with Tris-buffered saline containing 0.1% Tween-20 (TBST), membranes were incubated in the blocking buffer with a secondary antibody coupled to horseradish peroxidase for 1 h at room temperature. After being washed by TBST, signals were detected using iBright^™^ FL 1500 Imaging System (Thermo Fisher Scientific, USA). Protein expression was quantified by analyzing the densitometry of bands using ImageJ and normalized for Gapdh.

### RNA-sequencing (RNA-seq)

The total RNA from PMCs pretreated with 0 or 1 ng/ml TGF-β1was purified using the RNeasy Mini Kit (QIAGEN, cat. no. 74104). The RNA concentration and purity of each sample were determined by NanoDrop 2000 (Thermo Fisher, USA). The RNA integrity was then evaluated using Agilent bioanalyzer 2100 (Agilent, USA) with a threshold of RIN > 7.0. Approximately 5 μg of total RNA was depleted of ribosomal RNA using a Ribo-Zero^™^ rRNA Removal Kit (Illumina Inc, cat. no. RZNB1056) according to the manufacturer’s instructions. The remaining RNA was treated with Rnase R (Epicentre Inc., cat. no. RNR07250) to remove linear RNAs and enrich circRNAs. The enriched circRNAs were fragmented and then the double-strand cDNA was synthesized. End-polishing was performed, and the cDNA fragments were then ligated with adapters. The ligated cDNA was then subjected to universal PCR amplification to obtain a sufficient library for sequencing. Agilent bioanalyzer 2100 was used to evaluate the quality of the library, while the Illumina Hiseq 4000 (LC Bio, China) was used for the RNA-sequencing. The data were then assembled and annotated with corresponding transcript symbols. The differentially expressed circRNAs and mRNAs were screened using R software with the criteria set as | log2 (fold change) | > 1 and *p* < 0.05.

### RNA interference and overexpression

Recombinant circPhf21a overexpression (oe-circPhf21a) plasmid and matched negative control plasmid (oe-NC) were constructed by Shanghai Genechem Co.Ltd Biotech (Shanghai, China). Small interference RNA targeting backsplice region of circPhf21a (si-circPhf21a) for circPhf21a knockdown and matched negative control (si-NC) were purchased from Ribobio (Shanghai, China). Small interference RNA targeting Vegfa (si-Vegfa) for Vegfa knockdown and matched negative control (si-NC) were purchased from Ribobio (Shanghai, China). Chondrocytes were maintained in a 6-well plate in DMEM supplemented with 10% FBS and cultured until 50–70% confluent. The plasmids were transfected into PMCs at a final concentration of 1.6 ug/ml using Lipofectamine 2000 (Thermo Fisher, cat. no. 11668019) according to the manufacturer’s instructions. PMCs were transfected with siRNA at a final concentration of 20 nM using Lipofectamine 2000. The efficiencies of overexpression or knockdown were examined by RT-qPCR 48 h after transfection. After the confirmation of successful circPhf21a or Vegfa overexpression or knockdown, PCMs were harvested for subsequent experiments.

### Osteoarthritis mouse model

Adult male C57BL/6J mice aged 10 weeks were maintained under standard animal housing conditions. The lentiviruses containing shRNA that targeted the backsplice region of circPhf21a (sh-circPhf21a) were constructed by GenePharma (Shanghai, China). The experimental OA model was induced by destabilization of the medial meniscus (DMM) surgery as previously described [34]. Briefly, the right medial meniscotibial ligament was transected to destabilize the medial meniscus. Sham surgery was performed by only resecting the skin of the right knee joint. The mice were randomly divided into three groups: the sham group, the DMM + sh-NC (control lentivirus) group and the DMM + sh-circPhf21a group. A total of 5 ul solution containing sh-circPhf21a or control vector virus (approximately 10^6^ plaque-forming units) was slowly injected into the articular cavity using a 33-G needle with a microliter syringe. Lentivirus was injected into mouse knee joints 1 week after surgery and repeated 4 weeks after surgery. The right knee joints were harvested for further analysis 8 weeks after surgery.

### Hematoxylin and eosin (H&E), Safranin O staining and immunofluorescence

Eight weeks after surgery, the mice were sacrificed, and the knee joint samples were fixed with 4% paraformaldehyde (Solarbio, cat. no. P1110) for 24 h. The knee joint samples were then decalcified in EDTA-buffered saline solution (Solarbio, cat. no. E1171, pH = 7.2). The solution was changed every 3 days and the decalcification was completed within 4 weeks. The decalcified samples were embedded in a solution containing equal 30% sucrose and Optimal Cutting Temperature compound (O.C.T. Sakura, cat. no. 4583) for preparing frozen sections. The 10 µm thick sagittal-oriented of the knee joints medial compartment loading area were sectioned in – 20 °C freezing microtomes (HM525NX, Thermo Fisher Scientific) for the following histological analysis.

#### Hematoxylin and eosin (H&E) staining

H&E staining was conducted using HE staining Kit (Beyotime, cat. no. C0105S) according to the manufacturer’s instructions. In brief, the sections were rinsed gently in distilled water and stained with hematoxylin solution for 5 min, then washed in running tap water for 10 min. The sections were differentiated in 1% acid alcohol for 10 s and washed in running tap water for 10 min. The sections were counterstained in eosin solution for 30 s and washed in running rap water for 5 min. The sections were dehydrated and cleared with 95% ethyl alcohol, absolute ethyl alcohol and xylene twice for 2 min each time, then mounted with the resinous medium.

#### Safranin O and Fast Green Staining

Safranin O and Fast Green Staining was using Modified Safranine O-Fast Green FCF Cartilage Stain Kit (Solarbio, cat. no. G1371) according to the manufacturer’s protocols. Briefly, the sections were hydrated in distilled water, stained with Weigert’s Iron Hematoxylin for 5 min, then washed gently with distilled water, until no excess dye leached out of from the sections and differentiated in 1% Acid-Alcohol for 3 s. The sections were rinsed gently in distilled water and stained with 0.2% Fast Green for 5 min, then rinsed quickly with 1% acetic acid solution for 10 s and stained in 0.1% safranin O solution for 5 min. The sections were dehydrated and cleared with 95% ethyl alcohol, absolute ethyl alcohol and xylene twice for 2 min each time, then mounted with the resinous medium. Cartilage destruction of mouse knee joints was scored using the Osteoarthritis Research Society International (OARSI) guideline which contained both photograph reference and text instruction [35].

#### Immunofluorescence

The sections were washed gently twice with PBS, permeabilized with 0.2% Triton-X for 10 min, washed gently in PBS and blocked for 2 h at room temperature with the block solution (1% Bovine Serum Albumin). The sections were then incubated with the primary antibody in block solution overnight at 4 °C. The sections were washed gently with 0.2% Triton-X for 1 min to reduce the background and washed with PBS for 1 min. The sections were incubated with secondary antibody for 1 h at room temperature covered with aluminum foil to protect from light, then washed gently with 0.2% Triton-X for 1 min to reduce the background and washed twice gently with PBS for 5 min. The sections were then stained with DAPI solution (1:2000, Beyotime, cat. no. C1005) for 5 min, then washed in PBS with 3 changes and mounted in the resinous medium. The positive fluorescence  cells on the medial tibial plateau area were calculated.

### Statistical analysis

The data are represented as a mean ± standard deviation (SD). When data corresponded to the normal distribution, independent t-tests and one-way ANOVA were used in the current study. The significance was established as *p* < *0.05*. SPSS 20.0 (SPSS, Inc., Chicago, IL, USA) and GraphPad Prism 7.0 (GraphPad Software Inc., La Jolla, CA, USA) were used for statistical analysis.

## Results

### TGF-β1 promotes the proliferation and ECM anabolism of PMCs

**Fig. 1:**
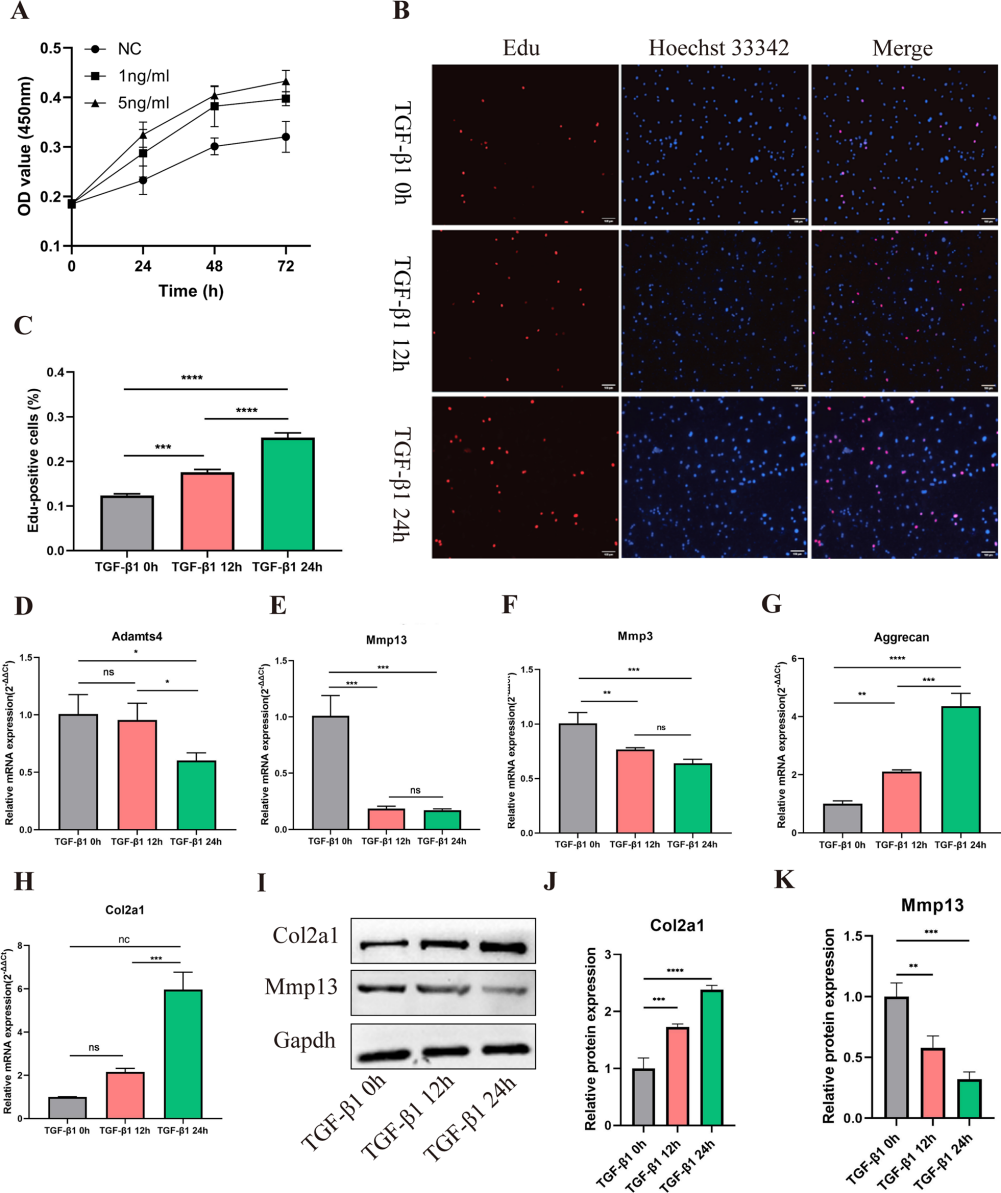
TGF-β1 promotes the proliferation and ECM anabolism of PMCs. **A** CCK-8 assay was used to determinate the proliferative capacities of PMCs. PMCs were treated with 0, 1 or 5 ng/ml TGF-β1 for 0 h, 24 h, 48 h or 72 h, respectively. Each experiment was performed in triplicate (n = 3). **B**, **C** EdU assay verified the proliferation capacity of PMCs treated with 1 ng/ml TGF-β1 for 0 h, 12 h or 24 h (n = 3), ***p < 0.001, ****p < 0.0001, scale bar = 100 μm. **D–H** mRNA levels of Adamts4, Mmp13, Mmp3, Aggrecan and Col2a1 were detected by RT-qPCR in PMCs treated with 1 ng/ml TGF-β1 for 0 h, 12 h or 24 h (n = 3), ns *p* > 0.05, *p < 0.05, **p < 0.01, ***p < 0.001, ****p < 0.0001. **I–K** Protein levels of Col2a1 and Mmp13 were analyzed using western blotting in PMCs treated with 1 ng/ml TGF-β1 for 0 h, 12 h or 24 h (n = 3). **p < 0.01, ***p < 0.001

PMCs were treated with 0, 1 or 5 ng/ml TGF-β1 respectively, and the cell viability was determined using CCK-8 assay. The OD value of the PMCs at 450 nm was collected after being treated with TGF-β1 for 0, 24, 48 or 72 h, respectively. Compared with PMCs without TGF-β1 treatment, those treated with 1 and 5 ng/ml TGF-β1 had higher OD values at 450 nm (Fig. 1A). Given that both 1 ng/ml and 5 ng/ml TGF-β1 treatments enhanced the proliferation of PMCs, we merely chose 1 ng/ml TGF-β1 for the following experiments. Consistently, the EdU assay showed that the proliferation ability of PMCs increased after being treated with 1 ng/ml TGF-β1 for 12 h and 24 h (Fig. 1B, C). We further explored the effect of TGF-β1 (1 ng/ml) on ECM metabolism at different treatment times. RT-qPCR assays indicated that the expression levels of Col2a1 and Aggrecan were significantly increased, while Mmp13, Mmp3 and Adamts4 were down-regulated after TGF-β1 treatment for 12 h or 24 h (Fig. 1D–H). Interestingly, no significant difference was identified in the expression level of Adamts4 after TGF-β1 treatment for 0 h and 12 h (Fig. 1D). Western blotting also showed that the protein level of Col2a1 was gradually increased, while Mmp13 was down-regulated after TGF-β1 treatment in a time-dependent manner (Fig. 1I–K). Taken together, these results indicated that TGF-β1 promoted the proliferation and ECM anabolism of PMCs.

### RNA-seq reveals the TGF-β1-regulated circRNAs in PMCs

**Fig. 2:**
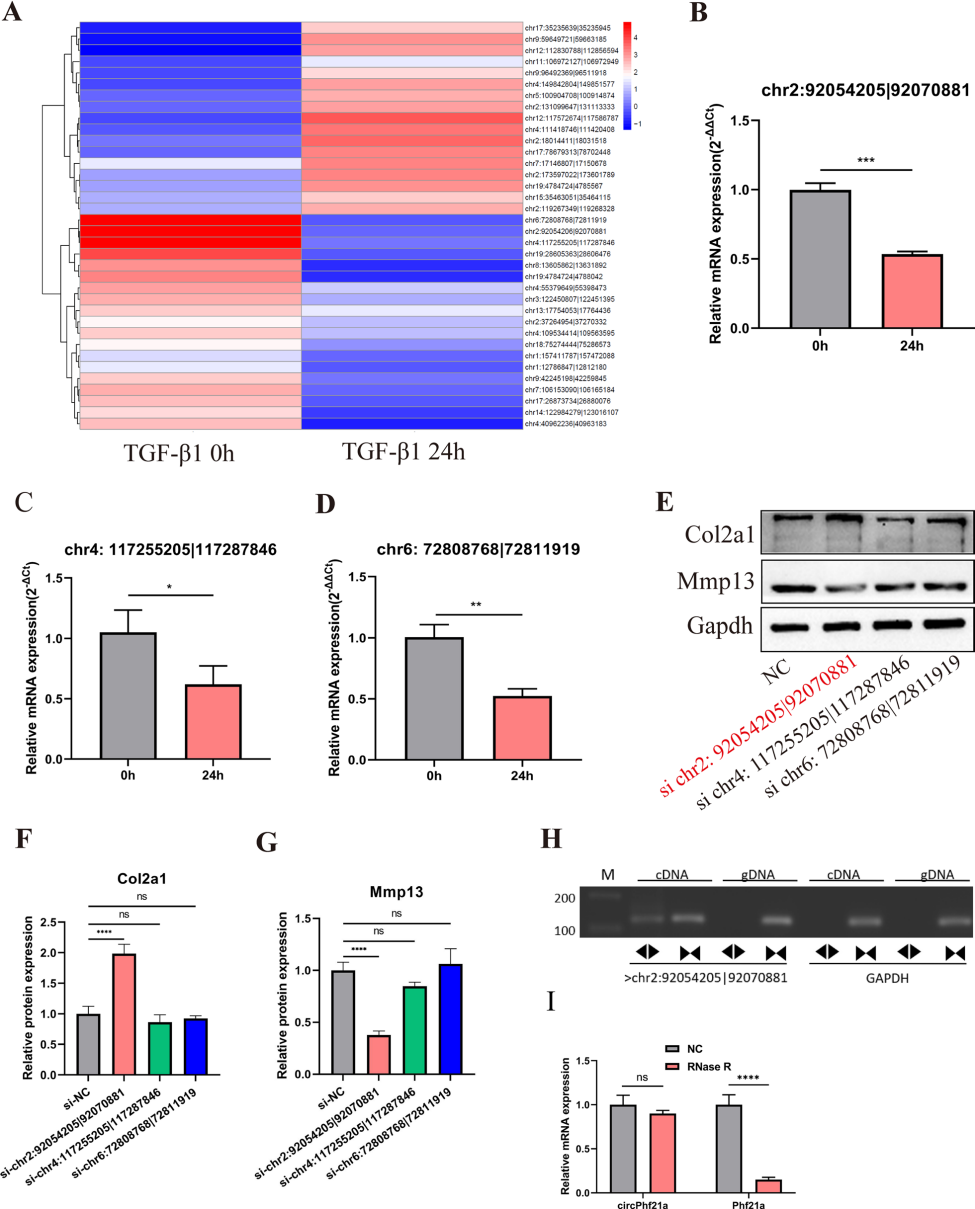
TGF-β1 related circRNA was identified by RNA-seq in PMCs. **A** Heatmap of all differentially expressed circRNAs, with | log2 (fold change) | > 1 and *p* < 0.05, between PMCs treated with 0 and 1 ng/ml TGF-β1 for 24 h. **B–D** Three most differentially expressed circRNAs were validated by RT-qPCR (n = 3, the results were consistent with those of sequencing), *p < 0.05, **p < 0.01, ***p < 0.001. **E–G** The protein expression levels of Col2a1 and Mmp13 were detected by western blotting after knockdown of chr2: 92054205|92070881 (circPhf21a), chr4: 117255205|117287846 and chr6: 72808768|72811919 in PMCs. (n = 3) ns *p* > 0.05, ***p < 0.001. **H** The presence of circPhf21a was validated in PMCs by RT-PCR. Divergent primers amplified circPhf21a from cDNA, but not from genomic DNA. Gapdh was used as a negative control. M = Marker. Back-to-back triangles represent divergent primers. Point-to-point triangles represent convergent primers. **I** The expression of circPhf21a and linear Phf21a mRNA in PMCs treated with or without RNase R was detected by RT-qPCR. The relative levels of circPhf21a and Phf21a mRNA were normalized to the values obtained with the mock treatment. n = 3 (three different experiments), ns *p* > 0.05, ****p < 0.0001

To gain insight into the TGF-β1 regulated circRNAs, we performed RNA-seq analysis of PMCs treated with 0 ng/ml and 1 ng/ml TGF-β1 for 24 h. We found that most of the circRNAs were in the length of 101–500 bp (Additional file 1: Fig. S1A). Most of the circRNAs in both groups originated from exons (Additional file 1: Fig. S1B). A total of 17 differentially expressed circRNAs were upregulated and 19 circRNAs were downregulated in the TGF-β1 treatment group (Fig. 2A, Additional file 1: Table S2). Interestingly, the differentially expressed circRNAs were located throughout the various chromosomes but not in chromosomes 10, 16, 20, 21, or 22 (Additional file 1: Fig. S1C).

### circPhf21a is downregulated in response to TGF-β1 treatment in PMCs

To further verify the results of RNA-seq, the three most differentially expressed circRNAs (chr6: 72808768|72811919, chr4: 117255205|117287846 and chr2: 92054205|92070881) were chosen for further experiments. Consistent with the results of RNA-seq, RT-qPCR indicated that three mentioned circRNAs were significantly down-regulated after TGF-β1 treatment (Fig. 2B–D). Moreover, western blotting for Col2a1 and Mmp13 expression reveals that knockdown chr2: 92054205|92070881 can promote ECM anabolism, which is derived from back splicing of Phf21a pre-mRNA (hereafter referred to as circPhf21a) (Fig. 2E–G). In the subsequent step, Sanger sequencing verified the back-splicing sites of the circRNAs (Additional file 1: Fig. S1D). Additionally, agarose gel electrophoresis revealed that circRNAs were amplified by divergent primers in cDNA but not in genomic DNA (Fig. 2H). Also, RNase R treatment was conducted to verify the stability of circPhf21a. The results showed that circPhf21a was resistant to RNase R, whereas its linear homologous transcript Phf21a mRNA level was significantly decreased after RNase R treatment (Fig. 2I). Collectively, these experimental results indicated that TGF-β1 regulated circRNA circPhf21a was cyclized in the molecule structure in PMCs.

### circPhf21a suppresses the proliferation and ECM anabolism of PMCs

**Fig. 3:**
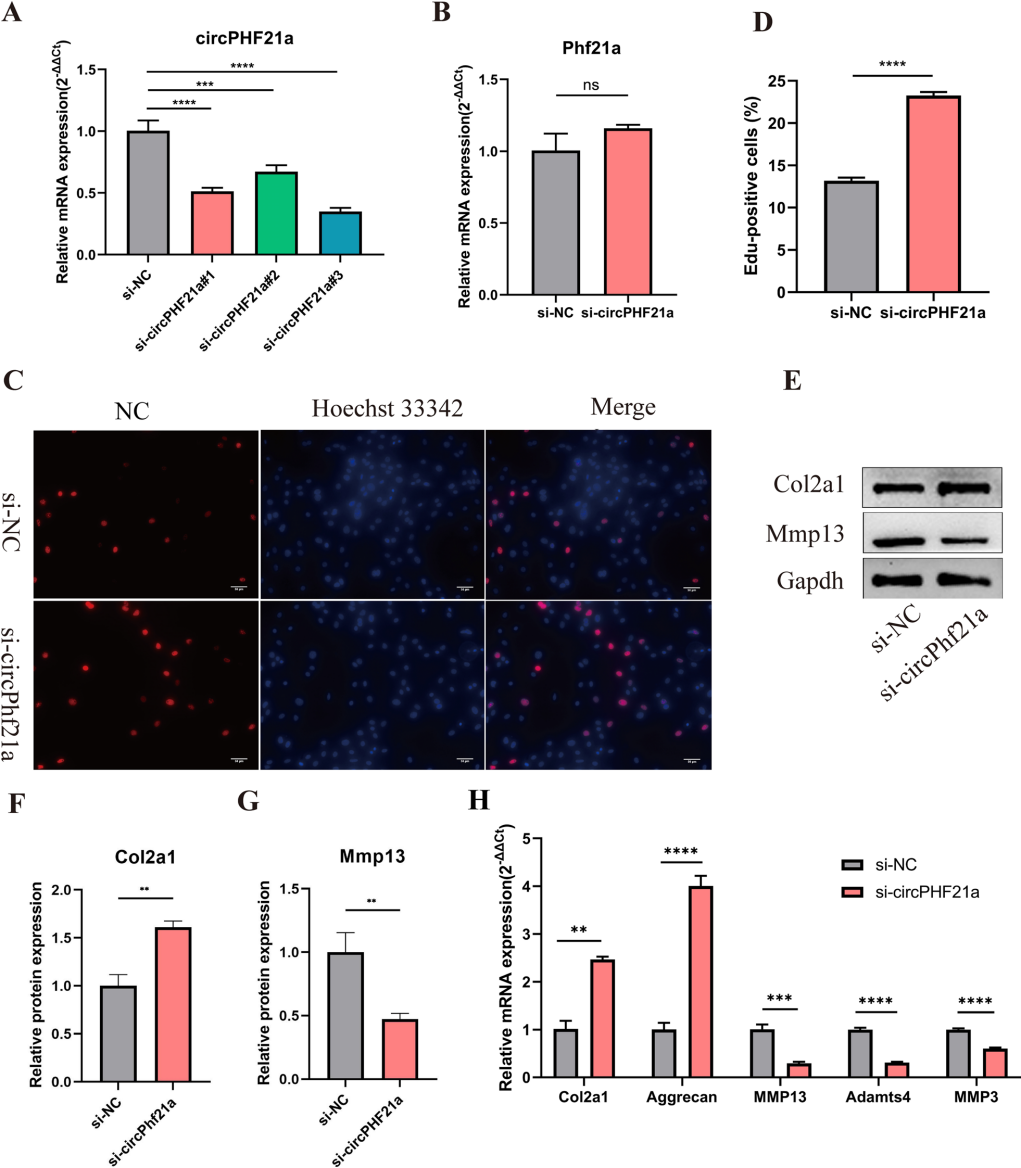
circPhf21a knockdown increased the proliferation and ECM anabolism of PMCs. **A** PMCs were transfected with circPhf21a siRNA or negative control siRNA at a final concentration of 20 nM. After 48 h of transfection, the expression level of circPhf21a was measured by RT-qPCR and normalized to Gapdh level. n = 3, ***p < 0.001, ****p < 0.0001. **B** PMCs were transfected with circPhf21a#3 siRNA (si-circPhf21a) or negative control siRNA (si-NC) at a final concentration of 20 nM. After 48 h of transfection, the expression level of Phf21a was measured by RT-qPCR and normalized to Gapdh level. n = 3, ns *p* > 0.05. **C**, **D** PMCs were transfected with si-circPhf21a or si-NC at a final concentration of 20 nM. After 48 h of transfection, the proliferation of PMCs was assessed using EdU assay. n = 3, ****p < 0.0001, scale bar = 50 μm. **E–G** PMCs were transfected with si-circPhf21a or si-NC at a final concentration of 20 nM. After 48 h, protein levels of Mmp13 and Col2a1 were determined by western blotting. n = 3, **p < 0.01. **H** PMCs were transfected with si-circPhf21a or si-NC at a final concentration of 20 nM. After 48 h, the mRNA expression levels of Mmp3, Mmp13, Adamts4, Col2a1 and Aggrecan were detected by RT-qPCR analysis in PMCs. n = 3, **p < 0.01, ***p < 0.001, ****p < 0.0001

**Fig. 4:**
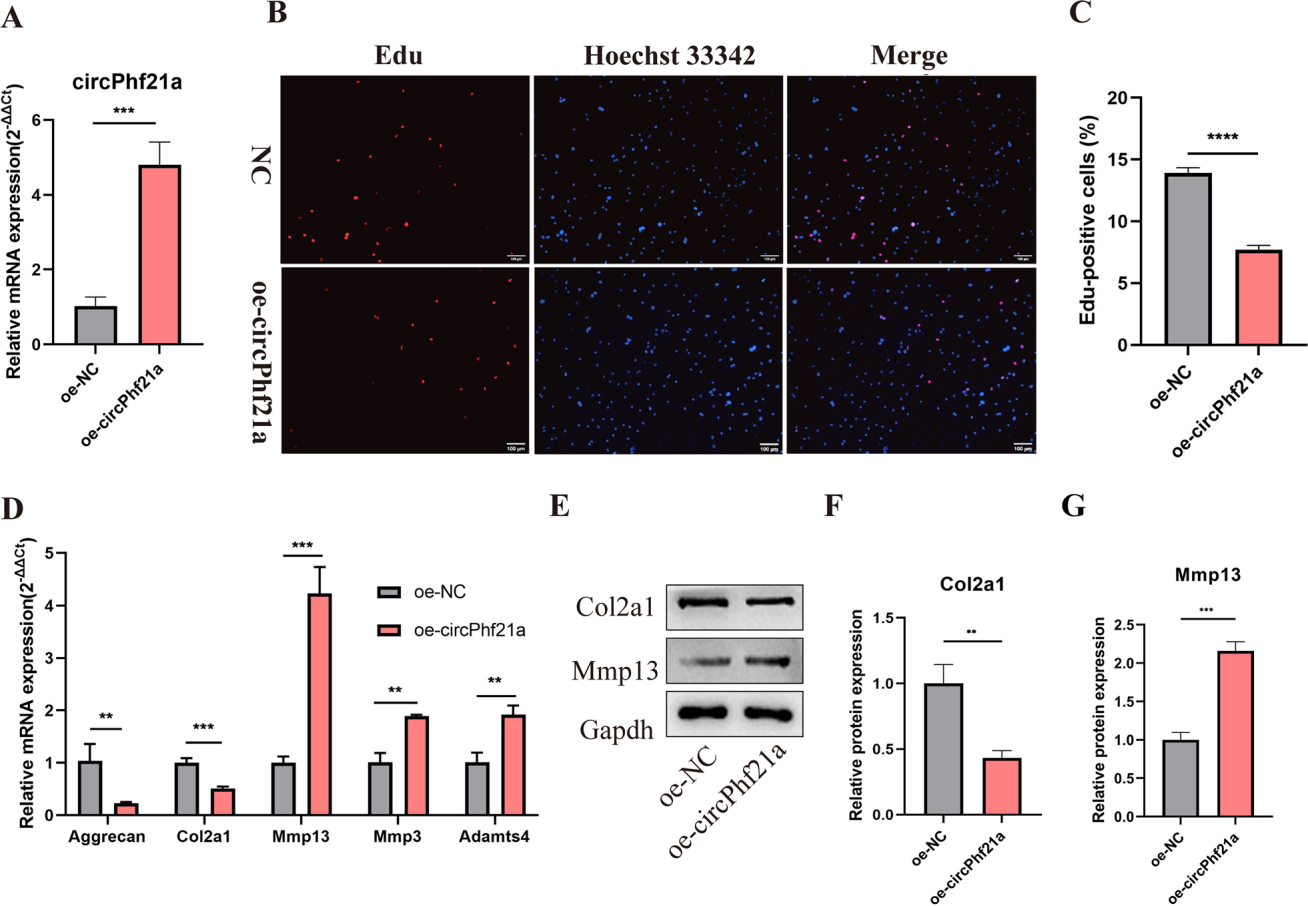
circPhf21a overexpression suppressed the proliferation and ECM anabolism of PMCs. **A** PMCs were transfected with circPhf21a overexpression plasmid (oe-circPhf21a) or negative control plasmid (oe-NC) at a final concentration of 1.6 ug/ml. After 48 h of transfection, the expression level of circPhf21a was measured by RT-qPCR and normalized to Gapdh level. n = 3, ***p < 0.001. **B**, **C** PMCs were transfected with oe-circPhf21a or oe-NC at a final concentration of 1.6 ug/ml. After 48 h of transfection, the proliferation of PMCs was assessed using EdU assay. n = 3, ****p < 0.0001, scale bar = 100 μm. **D** PMCs were transfected with oe-circPhf21a or oe-NC at a final concentration of 1.6 ug/ml. After 48 h, the mRNA expression levels of Mmp3, Mmp13, Adamts4, Col2a1 and Aggrecan were detected by RT-qPCR analysis in PMCs. n = 3, **p < 0.01, ***p < 0.001. **E-G** PMCs were transfected with oe-circPhf21a or oe-NC at a final concentration of 1.6 ug/ml. After 48 h, protein levels of Mmp13 and Col2a1 were determined by western blotting. n = 3, **p < 0.01

To further assess the biological functions of circPhf21a, we transfected PMCs with circPhf21a small-interfering RNA (si-circPhf21a). RT-qPCR revealed that the expression level of circPhf21a was significantly reduced after the PMCs were transfected with si-circPhf21a (Fig. 3A). Knockdown of circPhf21a expression did not affect Phf21a mRNA level (Fig. 3B). EdU assay indicted that silence of circPhf21a promoted the PMCs viability (Fig. 3C, D). Additionally, RT-qPCR and western blotting showed that inhibition of circPhf21a promoted the ECM anabolism of PMCs (Fig. 3E–H). Additionally, we prepared oe-circPhf21a plasmid to upregulate the expression of circPhf21a. The plasmid increased the level of circPhf21a approximately five times as compared with the control group (Fig. 4A). EdU assay indicated that upregulation of circPhf21a inhibited the viability of PMCs (Fig. 4B, C). Meanwhile, RT-qPCR and western blotting also showed that circPhf21a overexpression inhibited the ECM anabolism of PMCs (Fig. 4D–G). These results collectively demonstrated that circPhf21a suppressed the proliferation and ECM anabolism of PMCs.

### TGF-β1 regulates the proliferation and ECM anabolism of PMCs via circPhf21a

**Fig. 5:**
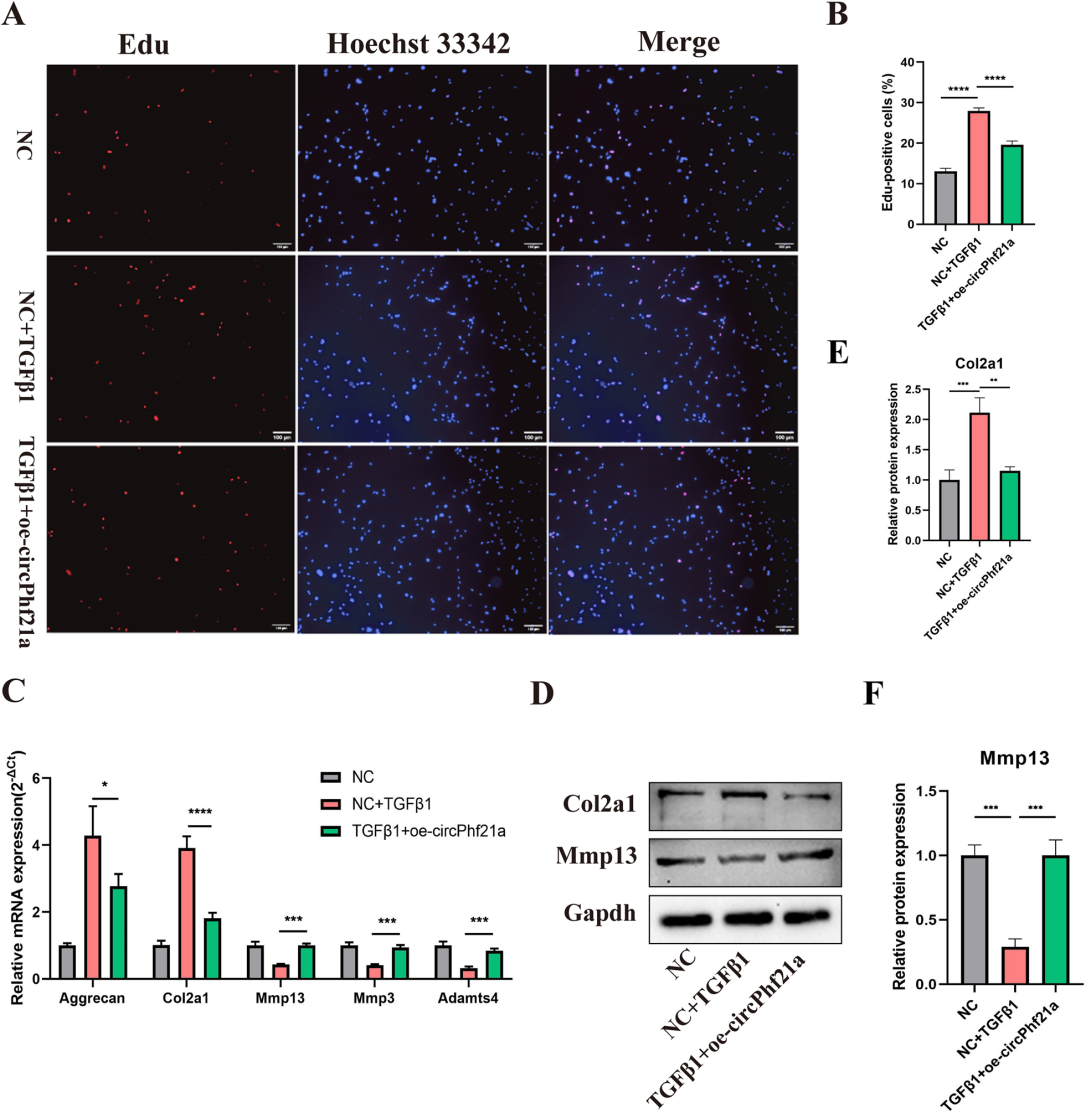
Overexpression of circPhf21a reversed TGF-β1 induced the proliferation and ECM anabolism of PMCs. **A**, **B** PMCs were incubated with negative control vector or TGF-β1 with or without oe-circPhf21a plasmid. After 48 h of incubation, the proliferation of PMCs was assessed using EdU assay. n = 3, ****p < 0.0001, scale bar: 100 μm. **C** PMCs were incubated with negative control vector or TGF-β1 with or without oe-circPhf21a. After 48 h of incubation, mRNA expression levels of Mmp13, Mmp3, Adamts4, Col2a1 and Aggrecan were detected by RT-qPCR analysis. n = 3, *p < 0.05, ***p < 0.001, ****p < 0.0001. **D–F** PMCs were incubated with negative control vector or TGF-β1 with or without oe-circPhf21a. After 48 h of incubation, the protein levels of Col2a1 and Mmp13 in PMCs were detected by western blotting. n = 3, **p < 0.01, ***p < 0.001

To examine whether TGF-β1 functions via regulating circPhf21a, we incubated PMCs with TGF-β1 (1 ng/ml) and oe-circPhf21a plasmid. As shown in Fig. 5A, B, TGF-β1 increased the proliferation rate of PMCs while this rate was markedly dampened by overexpression of circPhf21a. Moreover, RT-qPCR showed that mRNA expression levels of Mmp13, Mmp3 and Adamts4 significantly increased while those of Col2a1 and Aggrecan decreased in the cells co-incubated with TGF-β1 and oe-circPhf21a plasmid as compared with the cells incubated with TGF-β1 alone (Fig. 5C). Consistently, the protein levels of Col2a1 and Mmp13 showed similar results (Fig. 5D–F). Taken together, these results indicated that TGF-β1 functions by targeting circPhf21a in vitro.

### The regulation mechanism of circPhf21a was determined by RNA-sequencing and RT-qPCR

**Fig. 6:**
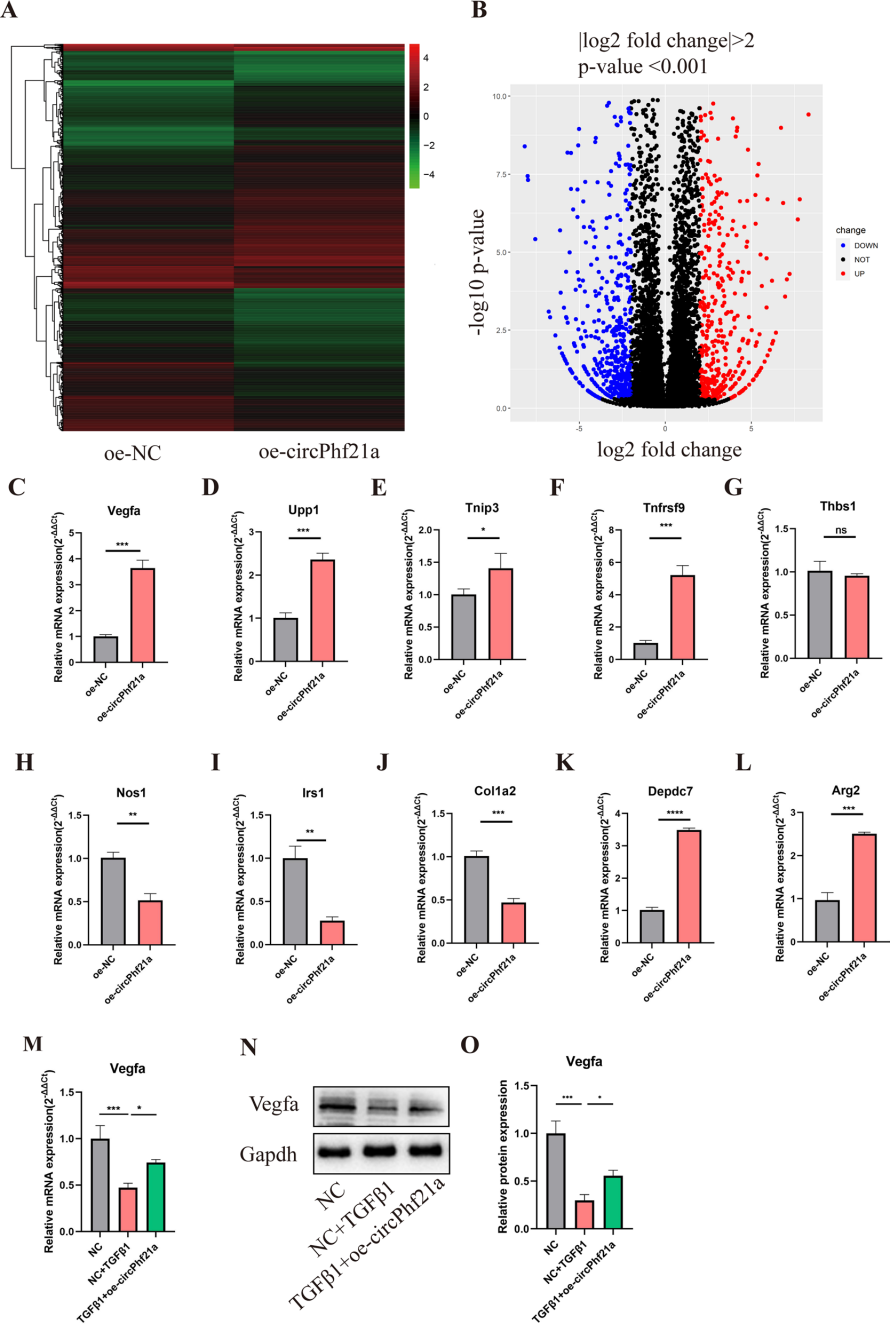
The regulation mechanism of circPhf21a was determined by RNA-sequencing and RT-qPCR. **A** Heatmap of the differentially expressed genes, with | log2 (fold change) | > 2 and *p* < 0.001, between PMCs transfected with oe-NC and oe-circPhf21a plasmid. PMCs transfected with oe-NC are the control for the experiment. Red rectangles represent high expression, and green rectangles represent low expression. **B** Volcano plot of the differentially expressed mRNAs between PMCs transfected with oe-NC and oe-circPhf21a plasmids. The red plots represent upregulated genes, the black plots represent nonsignificant genes, and the blue plots represent downregulated genes. **C–L** The mRNA expression levels of the 10 most differentially expressed genes were detected by RT-qPCR. n = 3, *p < 0.05, **p < 0.01, ***p < 0.001, ****p < 0.0001. **M**, **N** PMCs were incubated with negative control vector or TGF-β1 with or without oe-circPhf21a. After 48 h of incubation, mRNA and protein expression levels of Vegfa were detected by RT-qPCR or western blotting analysis. n = 3, *p < 0.05, ***p < 0.001

To further explore the mechanisms of circPhf21a regulating the PMCs proliferation and ECM anabolism, RNA-seq was conducted after overexpressing circPhf21a. A total of 933 differentially-expressed mRNAs (| log2 (fold change) | > 2 and *p* < 0.001) were identified between PMCs transfected with oe-NC and oe-circPhf21a plasmid. Of these, 398 mRNA were significantly upregulated and 535 downregulated (Fig. 6A, B; Additional file 1: Table S3). First, we choose the top ten differentially expressed genes. Furthermore, RT-qPCR verified that the expression level of 9 genes was consistent with the results of RNA-seq (Fig. 6C–L). Interestingly, of these differentially expressed genes, Vegfa has been verified to participate in cell proliferation and EMC metabolism [36–40]. Therefore, Vegfa was chosen for further investigation. To examine whether Vegfa was regulated by TGF-β1 via circPhf21a, we incubated PMCs with TGF-β1 (1 ng/ml) and overexpression of circPhf21a. As shown in Fig. 6M–O, TGF-β1 decreased the protein and mRNA level of Vegfa while this level was markedly increased by overexpression of circPhf21a.

### Vegfa knockdown elevated the proliferation and ECM anabolism of PMCs

**Fig. 7:**
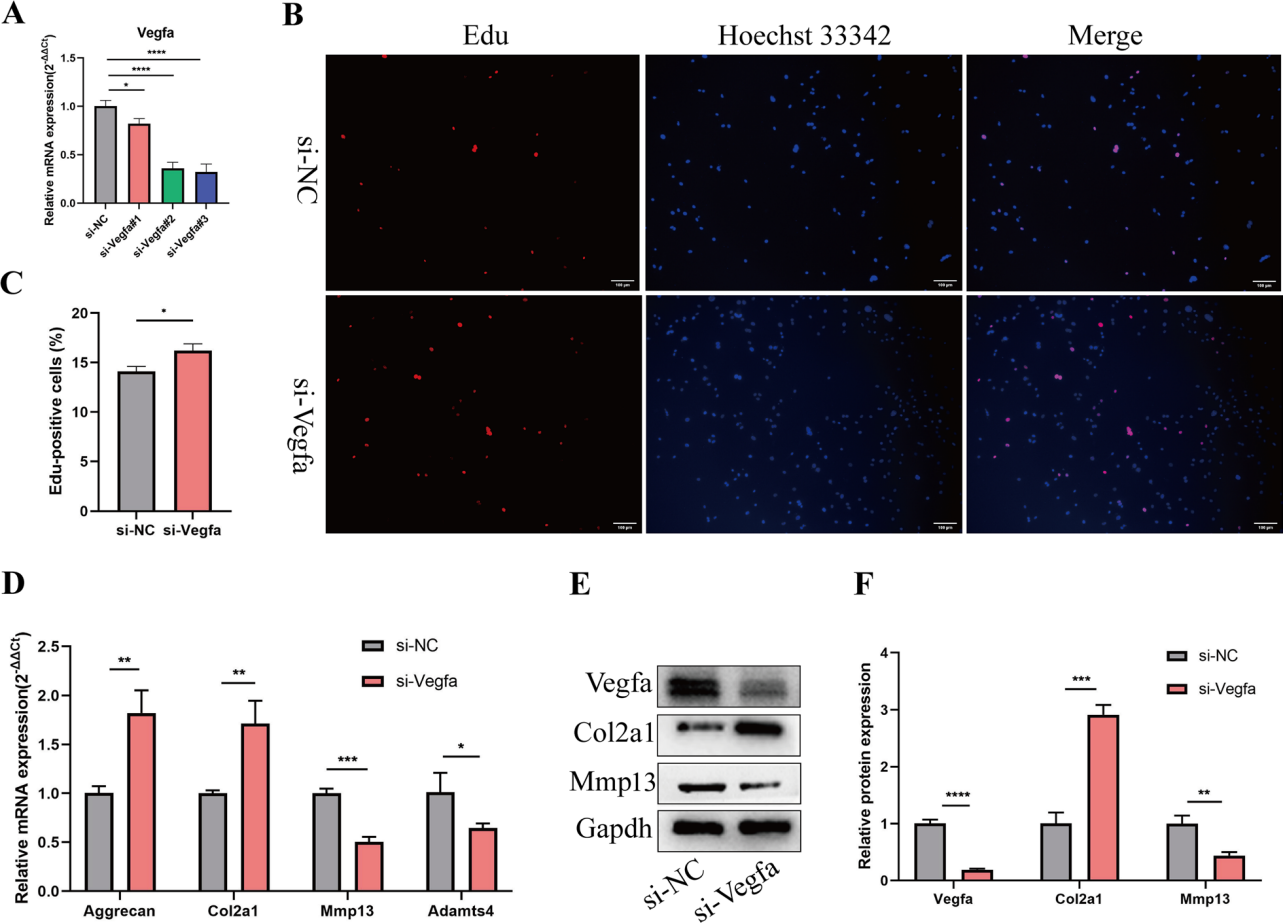
Vegfa knockdown increased the proliferation and ECM anabolism of PMCs. **A** PMCs were transfected with Vegfa siRNA or negative control siRNA at a final concentration of 20 nM. After 48 h of transfection, the expression level of Vegfa was measured by RT-qPCR and normalized to Gapdh level. n = 3, ***p < 0.001, ****p < 0.0001. **B**, **C** PMCs were transfected with Vegfa#3 siRNA (si-Vegfa) or negative control siRNA (si-NC) at a final concentration of 20 nM. After 48 h of transfection, the proliferation of PMCs was assessed using EdU assay. n = 3, ****p < 0.0001, scale bar = 100 μm. **D–F** PMCs were transfected with si-Vegfa or si-NC at a final concentration of 20 nM. After 48 h, protein levels of Mmp13 and Col2a1 as well as the mRNA expression levels of Mmp13, Adamts4, Col2a1 and Aggrecan were detected by western blotting or RT-qPCR analysis in PMCs. n = 3, *p < 0.05, **p < 0.01, ***p < 0.001, ****p < 0.0001

To assess the involvement of Vegfa in the regulation of proliferation and ECM anabolism of PMCs, we transfected PMCs with Vegfa small-interfering RNA (si-Vegfa). Three siRNAs were designed to knock down the expression of Vegfa. RT-qPCR showed that Vegfa siRNA#3 had a high knocking down efficiency, so it was chosen for subsequent experiments (Fig. 7A). EdU assay indicted that Vegfa knockdown promoted the PMCs viability (Fig. 7B, C). Additionally, RT-qPCR and western blotting showed that inhibition of Vegfa promoted the ECM anabolism of PMCs (Fig. 7D–F).

### circPhf21a suppresses the proliferation and ECM anabolism of PMCs via Vegfa

**Fig. 8:**
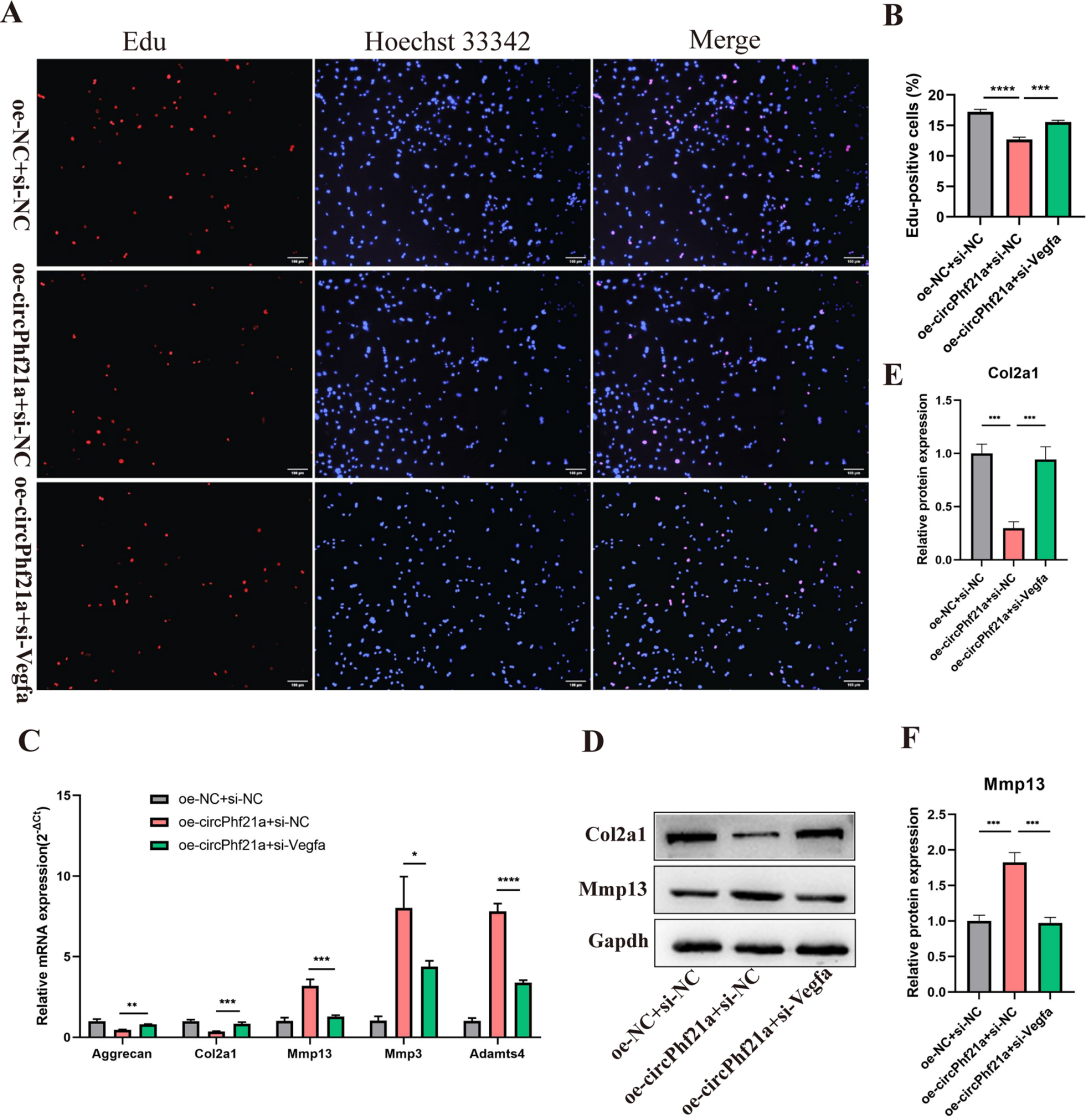
Knockdown of Vegfa reversed overexpression of circPhf21a induced ECM degradation and proliferation inhibition in PMCs. **A**, **B** PMCs were transfected with negative control vector or oe-circPhf21a with or without si-Vegfa. After 48 h of transfection, the proliferation rate of PMCs was detected by EdU assay. n = 3, ***p < 0.001, ****p < 0.0001, scale bar = 100 μm. **C** PMCs were transfected with negative control vector or oe-circPhf21a with or without si-Vegfa. After 48 h of transfection, mRNA expression levels of Mmp13, Mmp3, Adamts4, Col2a1 and Aggrecan were detected by RT-qPCR analysis. n = 3, *p < 0.05, **p < 0.01, ***p < 0.001, ****p < 0.0001. **D** PMCs were transfected with negative control vector or oe-circPhf21a with or without si-Vegfa. After 48 h of transfection, the protein levels of Col2a1 and Mmp13 in PMCs were detected by western blotting. n = 3, ***p < 0.001

We designed a rescue experiment to verify whether the effects of circPhf21a on chondrogenic phenotypes were achieved through Vegfa. The results showed that overexpression of circPhf21a decreased the proliferation of PMCs as well as decreased the expression levels of Col2a1 and Aggrecan and increased Mmp3, Mmp13 and Adamts4 levels in PMCs as compared with the negative control cells. However, these effects were abolished after Vegfa was knocked down (Fig. 8A–F). Taken together, these findings indicated that circPhf21a overexpression may suppress the proliferation and ECM anabolism of PMCs by targeting Vegfa in vitro.

### Silence of circPhf21a alleviated the progression of OA in a mouse model

**Fig. 9:**
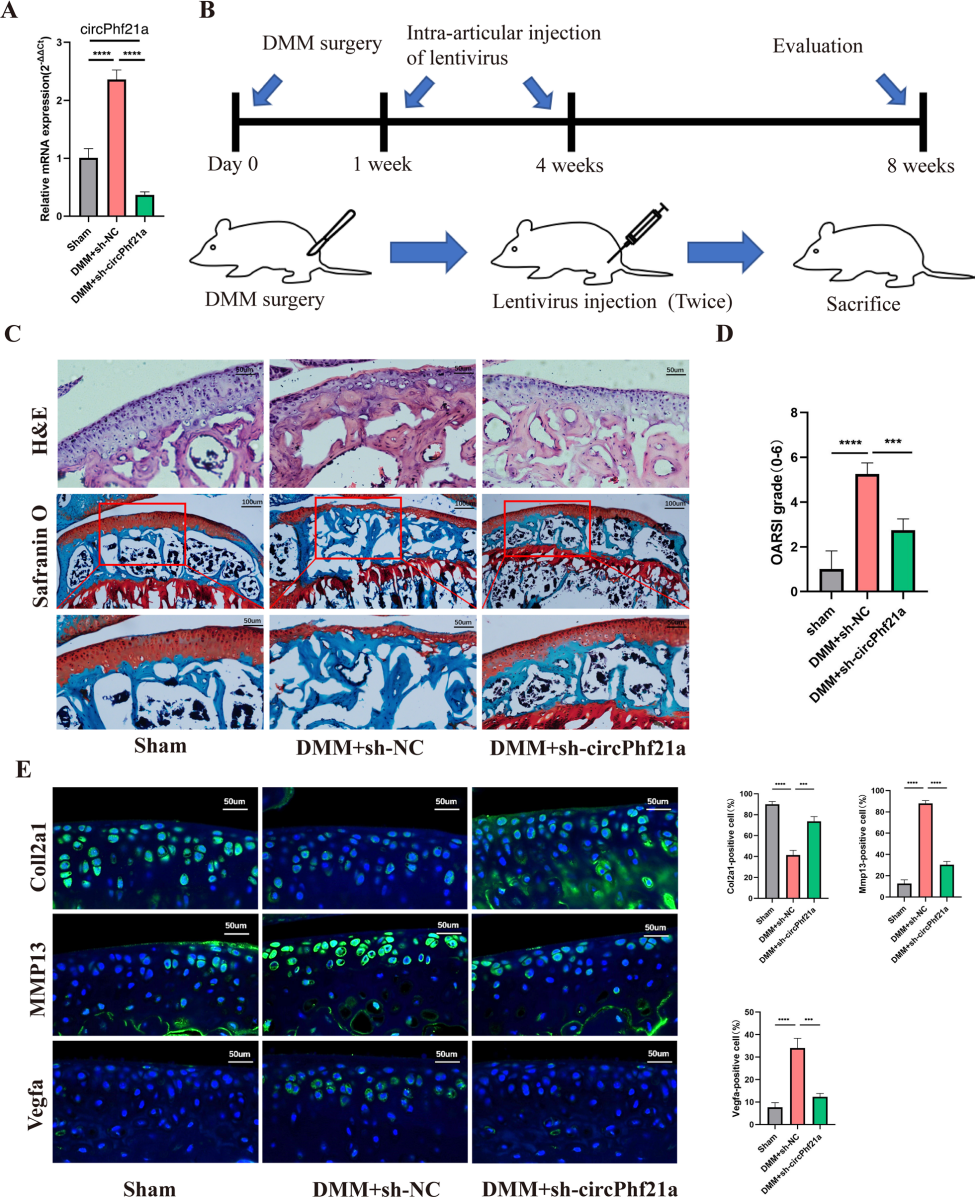
Silence of circPhf21a alleviated the progression of OA in mouse model. **A** RT-qPCR analysis of circPhf21a expression in knee articular cartilage from OA mice in different groups (n = 3), **p < 0.01, ****p < 0.0001. **B** Schematic of the time course used for the DMM-induced in vivo osteoarthritis experiments. **C** H&E staining was performed to observe the cell morphology and tissue integrity in the articular cartilage tissues of the mouse knee in different groups. The figures located at the top show the extent of damage and the morphology of chondrocytes in the contact area of the weight-bearing region between the medial tibial plateau and the medial femoral condyle. Likewise, the middle and bottom figures show Safranin O and fast green staining of articular cartilage tissues from mice that underwent sham or DMM surgery. Safranin O and fast green staining demonstrated OA progression through the 8-week time course in the medial tibial plateau. Scale bar = 50 or 100 μm. **D** OARSI scores of the medial tibial plateau of different group mice (Sham, n = 6; DMM + sh-NC, n = 6; DMM + sh-circPhf21a, n = 6). ***p < 0.001, ****p < 0.0001. **E** Representative images for Col2a1 (green), Mmp13 (green) and Vegfa (green) immunofluorescent staining in cartilage tissues obtained from sham or DMM mouse knees (Sham, n = 6; DMM + sh-NC, n = 6; DMM + sh-circPhf21a, n = 6). Scale bar = 50 μm. The bar graphs show quantification (%) of the Col2a1, Mmp13 or Vegfa positive cells from total cell population per field in immunofluorescent sections

The above findings revealed that circPhf21a, a TGF-β1 related circRNA, participated in regulating the proliferation and ECM anabolism of PMCs. It is well known that the progressive degradation of ECM is the hallmark event in the pathogenesis of OA [41, 42]. Accordingly, it aroused our great interest whether circPhf21a acted as a potential OA inducer in mouse model. To explore the potential role of circPhf21a in the progression of OA, we firstly examined the expression level of circPhf21a in the DMM induced OA mouse model. RT-qPCR revealed that circPhf21a was significantly upregulated in the DMM-induced OA mouse model (Fig. 9A). Furthermore, we inhibited the expression of circPhf21a through intra-articular injection of sh-circPhf21a (Fig. 9B). The silencing efficiency of circPhf21a was verified by RT-qPCR (Fig. 9A). H&E staining and safranin O and fast green staining showed that the thickness of the cartilage layer increased after the injection of sh-circPhf21a as compared with the DMM + sh-NC group (Fig. 9C). Quantitative analysis with OARSI scoring showed that circPhf21a knockdown significantly lowered OARSI scores as compared with the DMM + sh-NC group (Fig. 9D). Immunofluorescence showed that inhibition of circPhf21a increased the expression of Col2a1 and decreased the expression of Mmp13 and Vegfa as compared with the DMM + sh-NC group (Fig. 9E). Taken together, these findings demonstrated that circPhf21a was overexpressed in the DMM induced OA mouse model and silence of circPhf21a alleviated the progression of OA in vivo.

## Discussion

To date, whether and how circRNAs contribute to TGF-β1 induced proliferation and ECM anabolism of PMCs remains elusive. In the present study, we demonstrated that circPhf21a, a TGF-β1 related circRNA, participated in regulating the proliferation and ECM anabolism of PMCs through Vegfa. Furthermore, circPhf21a was significantly overexpressed in the mouse OA model and inhibition of circPhf21a significantly relieved the progression of OA.

Increasing evidence revealed that TGF-β1 signaling deregulation was associated with the onset and progression of OA [7]. Actually, the role of the TGF-β1 pathway as a cartilage-protector or destroyer is still controversial, owing to its pleiotropic effects at different stages of OA [7, 18]. Similarly, previous studies also found that the TGF-β1 signaling pathway plays a vital role in cancers [43, 44] and several drugs targeting the TGF-β1 pathway were developed in recent years [45, 46]. However, many clinical trials showed that some drugs targeting the TGF-β1 pathway for cancers treatment were ineffective, due to the dual roles of the TGF-β1 pathway in the suppression and promotion of cancers [47]. Accordingly, the routine clinical application of drugs targeting TGF-β1 may also be challenging owing to the dual roles and pleiotropic nature of the TGF-β1 during OA development, although the related clinical evidence was still lacking. Regardless of the dual roles of the TGF-β1 pathway in OA, it was essential to clarify the molecular mechanisms of the TGF-β1 pathway and related genes fulfilling their roles in OA, which may provide novel therapeutic targets for OA. Recently, researchers found that apart from coding RNAs, non-coding RNA (ncRNAs) also participated in the pathogenesis of OA [48, 49]. Of these ncRNAs, circRNAs play an increasingly important role in the pathogenesis of OA [23, 26, 50], but the potential roles of TGF-β1 related circRNAs in OA remain to be further clarified.

In our study, we firstly explored the effect of TGF-β1 on PMCs. We found that TGF-β1 promoted the proliferation and ECM anabolism of PMCs, which suggested that TGF-β1 may act as a chondroprotective factor under the current experimental condition. Moreover, we identified that circPhf21a, a TGF-β1 related circRNA, was significantly downregulated after being treated with TGF-β1. Additionally, suppression of circPhf21a promoted the proliferation and ECM anabolism of PMCs, whereas overexpression of circPhf21a had the opposite effects. These findings showed that circPhf21a was a cartilage destruction factor in the current experiments, which further drove us to explore the potential roles of circPhf21a in OA. Consistent with our hypothesis, we found that circPhf21a was significantly upregulated in the mouse OA model, and inhibition of circPhf21a significantly relieved the pathological progression of OA.

Furthermore, we explored the molecular targets of circPhf21a in regulating the proliferation and ECM anabolism in PMCs. RNA-seq analysis identified that Vegfa was differentially expressed after overexpressing circPhf21a. Vegfa, a member of the VEGF family, plays a critical role in regulating articular cartilage catabolism [51]. Integrated bioinformatics analysis also identified that VEGFA was a hub gene participating in the pathogenesis of OA [50, 52]. Previous studies also found that VEGFA was upregulated in OA articular cartilage, subchondral bone, synovium, synovial fluid and serum of OA patients [53–58]. Low-intensity pulsed ultrasound alleviated ECM degeneration by inhibiting Vegfa [59]. Ludin et al. showed that local intra-articular injection of VEGF in the knee joints of mice induced OA-like changes in the joint, including proteoglycan loss, calcification of the articular cartilage, cartilage degradation, bone sclerosis, osteophyte formation and synovial hyperplasia [60]. Moreover, in rabbit OA model, treatment with bevacizumab, an anti-VEGF antibody, increased aggrecan and type II collagen expression in the articular chondrocytes [40, 61]. Meng et al. demonstrated that VEGF silencing promoted cell proliferation and inhibited apoptosis in human chondrocyte cell line C28/I2 [39]. Gao et al. also found that silencing of VEGF decreased human chondrocytes cell apoptosis and induced cell division [62]. However, Chen et al. found that VEGFA was downregulated in OA chondrocytes, and overexpression of VEGFA in chondrocytes promoted cell proliferation and reduced matrix degradation [36]. Our findings indicated that inhibition of Vegfa promoted the proliferation and ECM anabolism of PMCs and the expression of Vegfa was positively correlated with the expression of circPhf21a. Collectively, our findings suggested that circPhf21a regulated the proliferation and ECM anabolism of PMCs via Vegfa.

A limitation must be admitted in the current study. We found that circPhf21a inhibited the proliferation and ECM anabolism of PMCs and acted as an inducer for OA development, but the specific interaction between circPhf21a and Vegfa was largely unclear. The detailed roles and mechanisms of circPhf21a remain to be further explored and may be published in a future paper.

To sum up, we found that TGF-β1 promoted the proliferation and ECM synthesis of PMCs. circPhf21a, a TGF-β1 related circRNA, participated in regulating the proliferation and ECM anabolism of PMCs through Vegfa. Furthermore, circPhf21a was significantly upregulated in the mouse OA model and inhibition of circPhf21a significantly attenuated the progression of OA. Our findings demonstrated that circPhf21a was involved in the pathogenesis of OA, which may provide novel therapeutic targets for OA treatment.

## Conclusions

We found that TGF-β1 promoted the proliferation and ECM synthesis of PMCs via the circPhf21a-Vegfa axis, which may provide novel therapeutic targets for OA treatment.

## Supplementary Information

**Additional file 1: Figure S1.** RNA-seq of the circRNAs for PMCs treated with and without TGF-β1.** Table S1**. Primers and sequences used in this study.** Table S2**. RNA-seq of differentially expressed circRNAs between PMCs with and without TGF-β1 treatment.** Table S3**. RNA-seq of differentially expressed mRNAs between PMCs transfected with oe-NC and oe-circPhf21a plasmids.

## Data Availability

Not applicable.
