# Supplementary material for: TGF-β1 regulates chondrocyte proliferation and extracellular matrix synthesis via circPhf21a-Vegfa axis in osteoarthritis
Source: Cell Commun Signal. 2022 May 30;20:75. doi: 10.1186/s12964-022-00881-9 (PMC9150374; doi:10.1186/s12964-022-00881-9)
Supplement: Supplementary file 2 — Additional file 1: Figure S1. RNA-seq of the circRNAs for PMCs treated with and without TGF-β1. Table S1. Primers and sequences used in this study. Table S2. RNA-seq of differentially expressed circRNAs between PMCs with and without TGF-β1 treatment. Table S3. RNA-seq of differentially expressed mRNAs between PMCs transfected with oe-NC and oe-circPhf21a plasmids. [file 12964_2022_881_MOESM2_ESM.docx]

**

**

**Supplementally Figure 1:** **RNA-seq of the circRNAs for PMCs after TGF-β1 treatment. (A)** Length of the circRNAs in the sequencing results. **(B)** Origin of the circRNAs. **(C)** The distribution of the differentially expressed circRNAs. **(D)** Schematic illustration showing Phf21a exons 2–6 circularization to form circPhf21a (black arrow). The presence of circPhf21a was validated by Sanger sequencing. The red arrow represents head-to-tail circPhf21a splicing sites.

| **Supplementary Table S1 Primers and sequences used in this study**   \|  \| \| \| \| --- \| --- \| --- \| \| Primers for qPCR \| \| \| \| Genes \| Primer sequences(5‘-3’） \|  \| \| Mmp3 \| F \| ACATGGAGACTTTGTCCCTTTTG \| \|  \| R \| TTGGCTGAGTGGTAGAGTCCC \| \| Mmp13 \| F \| CTTCTTCTTGTTGAGCTGGACTC \| \|  \| R \| CTGTGGAGGTCACTGTAGACT \| \| Adamts4 \| F \| ATGGCCTCAATCCATCCCAG \| \|  \| R \| AAGCAGGGTTGGAATCTTTGC \| \| Col2a1 \| F \| TGAAGACCCAGACTGCCTCAA \| \|  \| R \| AGCCGCGAAGTTCTTTTCTCC \| \| Aggrecan \| F \| CCTGCTACTTCATCGACCCC \| \|  \| R \| AGATGCTGTTGACTCGAACCT \| \| Gapdh \| F \| TGCACCACCAACTGCTTAG \| \|  \| R \| GGATGCAGGGATGATGTTC \| \| circPhf21a \| F \| AGGATCCACAGAATGCTGAC \| \|  \| R \| GGTTCTGAGAAGTCCAATTCTG \| \| Phf21a \| F \| GGGGACAGGGGCTGTAGTT \| \|  \| R \| CACCATGACAGGTCCTCGT \| \| Vegfa \| F \| CTGCCGTCCGATTGAGACC \| \|  \| R \| CCCCTCCTTGTACCACTGTC \| \| Upp1 \| F \| ACAGGAACTGAAGCAAAGGAC \| \|  \| R \| GTTGAAATGGTAGAGCACGTCTT \| \| Arg2 \| F \| TCCTCCACGGGCAAATTCC \| \|  \| R \| GCTGGACCATATTCCACTCCTA \| \| Depdc7 \| F \| ACAGAGACCTCCGGGTTTCA \| \|  \| R \| TGGTGCCTTCGCTTCTTCAC \| \| Tnfrsf9 \| F \| CCTTGCAGGTCCTTACCTTGT \| \|  \| R \| GTTGCTTGAATATGTGGGGGA \| \| Tnip3 \| F \| CTGGATGTCAATCAACAATGGGA \| \|  \| R \| ACTAGGGTGTGAGTGTTTTCTGT \| \| Col1a2 \| F \| GTAACTTCGTGCCTAGCAACA \| \|  \| R \| CCTTTGTCAGAATACTGAGCAGC \| \| Irs1 \| F \| CTCCTGCTAACATCCACCTTG \| \|  \| R \| AGCTCGCTAACTGAGATAGTCAT \| \| Thbs1 \| F \| GGGGAGATAACGGTGTGTTTG \| \|  \| R \| CGGGGATCAGGTTGGCATT \| \| Nos2 \| F \| GTTCTCAGCCCAACAATACAAGA \| \|  \| R \| GTGGACGGGTCGATGTCAC \| \| chr4:117255205\|117287846 \| F \| AGTGCTGGAGAGGGTCGAT \| \|  \| R \| GATGGGGAACTCAATGATTTCCTTT \| \| chr6:72808768\|72811919 \| F \| TGGGCTACACGGAAACATCT \| \|  \| R \| CTGCGACCTCGAAAATTTCCTT \| \|  \| \| \| \| shRNA \| \| \| \| circPhf21a \|  \| AGUGAGAAACAGGGGACUGGA \| \|  \| \| \| \| siRNA \| \| \| \| circPhf21a si-1 \|  \| UGAGAAACAGGGGACUGGAGA \| \| circPhf21a si-2 \|  \| AGAAACAGGGGACUGGAGAGC \| \| circPhf21a si-3 \|  \| AGUGAGAAACAGGGGACUGGA \| \| si-Vegfa si-1 \|  \| TGCGGATCAAACCTCACCAAA \| \| si-Vegfa si-2 \|  \| GATCAAGTTCATGGATGTCTA \| \| si-Vegfa si-3 \|  \| CGAGATAGAGTACATCTTCAA \| |
| --- | --- | --- | --- | --- | --- | --- | --- | --- | --- | --- | --- | --- | --- | --- | --- | --- | --- | --- | --- | --- | --- | --- | --- | --- | --- | --- | --- | --- | --- | --- | --- | --- | --- | --- | --- | --- | --- | --- | --- | --- | --- | --- | --- | --- | --- | --- | --- | --- | --- | --- | --- | --- | --- | --- | --- | --- | --- | --- | --- | --- | --- | --- | --- | --- | --- | --- | --- | --- | --- | --- | --- | --- | --- | --- | --- | --- | --- | --- | --- | --- | --- | --- | --- | --- | --- | --- | --- | --- | --- | --- | --- | --- | --- | --- | --- | --- | --- | --- | --- | --- | --- | --- | --- | --- | --- | --- | --- | --- | --- | --- | --- | --- | --- | --- | --- | --- | --- | --- | --- | --- | --- | --- | --- | --- | --- | --- | --- | --- | --- | --- | --- | --- | --- | --- | --- | --- | --- | --- | --- | --- | --- | --- | --- | --- | --- | --- | --- | --- | --- | --- | --- | --- | --- | --- | --- | --- | --- | --- | --- | --- | --- | --- |

Supplementary figure 2

| ID | TGFβ0h | TGFβ24h |
| --- | --- | --- |
| chr2:173597022\|173601789 | 0.65 | 3.16 |
| chr12:117572674\|117586787 | -0.32 | 3.69 |
| chr7:17146807\|17150678 | 1.52 | 3.26 |
| chr2:18014411\|18031518 | 0.19 | 3.58 |
| chr15:35463051\|35464115 | 0.85 | 2.44 |
| chr11:106972127\|106972949 | -0.4 | 1.59 |
| chr4:111418746\|111420408 | -0.19 | 3.5 |
| chr5:100904708\|100914874 | -0.09 | 2.76 |
| chr17:35235639\|35235945 | -0.99 | 2.4 |
| chr2:131099647\|131113333 | -0.02 | 2.85 |
| chr4:149842804\|149851577 | -0.32 | 2.87 |
| chr19:4784724\|4785567 | 0.63 | 3.06 |
| chr9:59649721\|59663185 | -1.03 | 3 |
| chr17:78679313\|78702448 | -0.03 | 3.28 |
| chr12:112830788\|112856594 | -1.34 | 2.91 |
| chr9:96492369\|96511918 | -0.36 | 2.28 |
| chr2:119267349\|119268328 | 0.74 | 2.69 |
| chr14:122984279\|123016107 | 2.21 | -0.62 |
| chr13:17754053\|17764436 | 2.35 | 1.57 |
| chr2:37264954\|37270332 | 1.87 | 0.97 |
| chr1:157411787\|157472088 | 1.32 | -0.01 |
| chr4:109534414\|109563595 | 2.32 | 1.03 |
| chr8:13605862\|13631892 | 3.01 | -0.67 |
| chr7:106153090\|106165184 | 2.68 | -0.02 |
| chr1:12786847\|12812180 | 1.55 | -0.14 |
| chr9:42245198\|42259845 | 2.31 | 0.11 |
| chr18:75274444\|75286573 | 1.86 | 0.46 |
| chr4:40962236\|40963183 | 2.63 | -0.87 |
| chr2:92054206\|92070881 | 4.76 | 0.02 |
| chr17:26873734\|26880076 | 2.48 | -0.14 |
| chr4:117255205\|117287846 | 4.84 | 0.15 |
| chr4:55379649\|55398473 | 2.84 | 1.21 |
| chr6:72808768\|72811919 | 4.89 | -0.16 |
| chr19:28605363\|28606476 | 3.9 | -0.24 |
| chr19:4784724\|4788042 | 3.3 | -0.75 |
| chr3:122450807\|122451395 | 2.75 | 0.84 |

Supplementary Table S3

| gene | oe-NC | oe-circPhf21a | Log2FC(oe-circPhf21a/NC) | P-value |
| --- | --- | --- | --- | --- |
| Upp1 | 0.06379385 | 20.22800223 | 8.30872073768179 | 7.43E-95 |
| Vegfa | 0.08807209 | 19.58706878 | 7.79700097370719 | 1.11E-67 |
| Arg2 | 0.07806531 | 16.07451934 | 7.68587835336644 | 2.93E-61 |
| Depdc7 | 0.06427573 | 9.50689731 | 7.20855657579204 | 8.90E-44 |
| Tnfrsf9 | 0.05533694 | 7.366329853 | 7.05655937342627 | 4.95E-42 |
| Tnip3 | 0.02934957 | 3.631717632 | 6.95116873277933 | 1.65E-36 |
| Cxcl3 | 0.21732522 | 24.89595615 | 6.83991198255325 | 1.72E-66 |
| Slit1 | 0.06335541 | 6.655494798 | 6.71493438707606 | 1.26E-90 |
| Grhl3 | 0.03980516 | 3.386276118 | 6.41060035171341 | 6.44E-25 |
| Camp | 0.18467202 | 14.10355223 | 6.25494948132835 | 2.55E-22 |
| Syt5 | 0.06321059 | 4.644123774 | 6.19909624668928 | 1.87E-21 |
| Rik | 0.02792692 | 2.038310546 | 6.18957347252136 | 1.30E-41 |
| Rab33a | 0.0948701 | 6.3281794 | 6.05969318991572 | 1.94E-19 |
| Jag2 | 0.10914508 | 6.647296317 | 5.92844865662717 | 1.02E-67 |
| Gipr | 0.0617291 | 3.759506036 | 5.92844865662717 | 1.03E-17 |
| Ascl2 | 0.13724385 | 8.026913357 | 5.87003197033529 | 5.46E-33 |
| Bdkrb1 | 0.24802363 | 14.46607471 | 5.86605211940162 | 8.68E-49 |
| Ltb4r2 | 0.07687181 | 4.161553435 | 5.7585236553033 | 1.05E-15 |
| Hs3st1 | 0.19694695 | 9.519619637 | 5.59502492302042 | 6.39E-40 |
| Muc2 | 0.01642687 | 0.7781283 | 5.56587857720862 | 1.05E-13 |
| Nipal4 | 0.13428654 | 6.166325193 | 5.5210243414981 | 5.47E-50 |
| Npas1 | 0.05290222 | 2.35251305 | 5.47473068930271 | 7.54E-13 |
| Dpp6 | 0.0454659 | 1.999850496 | 5.45896337336239 | 2.04E-24 |
| Cutal | 0.04703169 | 2.000523242 | 5.41060035188299 | 2.80E-12 |
| Rasd2 | 0.03936603 | 1.674459312 | 5.41060035188299 | 2.80E-12 |
| Slc4a11 | 0.24190246 | 10.22266184 | 5.40120165363689 | 5.25E-79 |
| Lilrb4a | 0.51621985 | 20.8883725 | 5.338570665483 | 2.57E-75 |
| Acer2 | 0.05260035 | 2.084840458 | 5.30872073768178 | 7.36E-22 |
| Gna15 | 0.05640925 | 2.235807998 | 5.30872073768178 | 1.99E-11 |
| Serpina3i | 0.05044165 | 1.950516381 | 5.27309682813306 | 3.83E-11 |
| Areg | 0.09104407 | 3.520561665 | 5.27309682813306 | 3.83E-11 |
| Fam110c | 0.24858099 | 9.372012052 | 5.23657095191662 | 6.92E-60 |
| Crct1 | 0.30855938 | 11.6333204 | 5.23657095191662 | 1.00E-20 |
| Scn3b | 0.18258189 | 6.833280693 | 5.22596285245936 | 4.36E-69 |
| Rtn4rl2 | 0.08758396 | 3.048087238 | 5.12109373468801 | 5.19E-10 |
| Chrm3 | 0.03491747 | 1.181438485 | 5.08045174997747 | 9.95E-10 |
| Smtnl2 | 0.12646937 | 4.075345297 | 5.01006242215003 | 9.58E-26 |
| Erich2 | 0.06324674 | 1.834254899 | 4.85805932885421 | 2.54E-08 |
| Hist1h1b | 0.13931806 | 4.040443095 | 4.85805932885421 | 2.54E-08 |
| Cox7a1 | 1.20894578 | 34.76915358 | 4.84598649649093 | 6.39E-30 |
| Gls2 | 0.26665779 | 7.647568013 | 4.8419396634071 | 3.94E-44 |
| Robo3 | 0.04681276 | 1.335016203 | 4.83381178248106 | 2.24E-15 |
| Bdkrb2 | 0.19753311 | 5.601465078 | 4.82563785099225 | 2.22E-22 |
| Tbc1d8 | 0.29910376 | 8.288947463 | 4.79247098687958 | 1.40E-83 |
| Kcna1 | 0.03699617 | 1.013338924 | 4.77559716875003 | 1.53E-21 |
| Gpr35 | 0.20905937 | 5.6083221 | 4.74558459952858 | 3.94E-54 |
| Pim2 | 0.54198206 | 14.51326104 | 4.74298280421896 | 3.45E-67 |
| Mreg | 0.35497325 | 9.265304889 | 4.7060562351328 | 1.84E-52 |
| Tspan33 | 0.05528163 | 1.389487044 | 4.65160845138679 | 3.35E-07 |
| Fam196a | 0.14313376 | 3.528438704 | 4.62359407507088 | 3.61E-37 |
| Mmp12 | 0.3066774 | 7.322831707 | 4.57760787003364 | 7.36E-59 |
| Tgm1 | 0.07757261 | 1.837277689 | 4.56587857751319 | 1.37E-12 |
| Ckmt1 | 0.13923038 | 3.23032027 | 4.53613123396685 | 2.60E-12 |
| Rab39 | 0.03723276 | 0.863847006 | 4.53613123396685 | 1.20E-06 |
| Fgf9 | 0.10690364 | 2.428626165 | 4.50575758476457 | 5.71E-23 |
| Slc10a6 | 0.10416058 | 2.366309721 | 4.50575758476457 | 4.91E-12 |
| Rgcc | 0.96821478 | 21.8788078 | 4.49806319325304 | 1.99E-44 |
| Eid3 | 0.33932067 | 7.544639244 | 4.47473068897828 | 2.03E-22 |
| Lingo3 | 0.06436924 | 1.431220479 | 4.47473068897828 | 9.28E-12 |
| Olr1 | 0.03100295 | 0.689336433 | 4.47473068897828 | 2.28E-06 |
| Sp6 | 0.09832759 | 2.154583739 | 4.45366907399434 | 7.99E-17 |
| Mab21l3 | 0.24872072 | 5.379913953 | 4.43498451030152 | 3.18E-42 |
| Celsr3 | 0.07663881 | 1.629940475 | 4.41060035188299 | 2.12E-41 |
| Tubb3 | 0.06420113 | 1.365418069 | 4.41060035188299 | 4.32E-06 |
| Dhh | 0.04673365 | 0.993922828 | 4.41060035188299 | 4.32E-06 |
| Gjc2 | 0.15146308 | 3.074867557 | 4.34348615566913 | 1.89E-15 |
| Srcin1 | 0.01588206 | 0.322423298 | 4.34348615566913 | 8.15E-06 |
| Nefh | 0.02771006 | 0.562544657 | 4.34348615566913 | 8.15E-06 |
| Ces2e | 0.57379013 | 11.56933001 | 4.33363837017033 | 4.86E-67 |
| Myot | 0.2004867 | 3.973193914 | 4.30872073804577 | 1.14E-19 |
| Tuba8 | 0.15449517 | 3.06174545 | 4.30872073804577 | 2.21E-10 |
| Pvrl4 | 0.12627687 | 2.441485402 | 4.27309682813306 | 4.01E-19 |
| H2-Q7 | 0.14403456 | 2.784819286 | 4.27309682813306 | 4.15E-10 |
| Slc5a8 | 0.02075005 | 0.401189498 | 4.27309682813306 | 1.54E-05 |
| Slc6a12 | 0.04315979 | 0.834467894 | 4.27309682813306 | 1.54E-05 |
| Mal2 | 0.03986254 | 0.770717554 | 4.27309682813306 | 1.54E-05 |
| Lilr4b | 0.22317123 | 4.242963399 | 4.24884928175991 | 2.36E-14 |
| Kcnj4 | 0.56542934 | 10.43530796 | 4.2059826319192 | 1.13E-47 |
| Has1 | 0.37013852 | 6.747462714 | 4.1882079306596 | 2.25E-30 |
| Wnt9a | 0.73345626 | 13.1495843 | 4.16416245679936 | 1.18E-90 |
| Cyfip2 | 0.36540659 | 6.486872744 | 4.14994859750795 | 1.43E-89 |
| Pgf | 0.96851754 | 16.85312024 | 4.12109373468801 | 3.73E-56 |
| Cyyr1 | 0.10882296 | 1.841021702 | 4.08045174997747 | 9.56E-09 |
| Svop | 0.79856447 | 13.45943544 | 4.07506520355056 | 8.43E-88 |
| Trpm2 | 0.04550955 | 0.762579148 | 4.06664595037864 | 1.89E-12 |
| Glt1d1 | 0.21286441 | 3.566855132 | 4.06664595037864 | 1.89E-12 |
| Gpr84 | 0.14264157 | 2.344203778 | 4.0386315740571 | 1.78E-08 |
| Bhlha15 | 0.03165957 | 0.520300524 | 4.0386315740571 | 0.000102 |
| Gm1045 | 0.14574248 | 2.39516473 | 4.0386315740571 | 0.000102 |
| Rnf208 | 0.8298465 | 13.55766185 | 4.03012007468467 | 1.55E-37 |
| Bmp8a | 0.32236877 | 5.253361011 | 4.0264568606764 | 1.38E-26 |
| Kcnn3 | 0.1016581 | 1.656632946 | 4.0264568606764 | 1.38E-26 |
| Atp7b | 0.18784723 | 3.019025485 | 4.00645116857857 | 1.06E-29 |
| Pkp2 | 0.30610457 | 4.845643354 | 3.98459173449834 | 3.65E-29 |
| Urah | 0.16387932 | 2.534804399 | 3.9511687332457 | 0.00019 |
| Ptprn2 | 0.02359611 | 0.364972903 | 3.9511687332457 | 0.00019 |
| St14 | 0.4956768 | 7.533778478 | 3.92590197450343 | 9.22E-61 |
| Mcpt8 | 3.69167949 | 56.08141437 | 3.92517352498409 | 1.27E-93 |
| Syt17 | 0.88932654 | 13.49113195 | 3.92315435685663 | 3.55E-44 |
| Tmeff2 | 0.09938773 | 1.505253623 | 3.92079508404341 | 4.19E-11 |
| Etnk2 | 0.14561458 | 2.205371587 | 3.92079508404341 | 4.19E-11 |
| Pglyrp1 | 0.32487089 | 4.867913184 | 3.90536504339187 | 1.15E-07 |
| Krt19 | 1.45020663 | 21.262795 | 3.87400087231325 | 4.29E-58 |
| Sag | 0.06438797 | 0.933676315 | 3.85805932835676 | 0.000354 |
| Fam222a | 0.04107632 | 0.59563903 | 3.85805932835676 | 0.000354 |
| Kcnj2 | 0.20304431 | 2.885416282 | 3.82891298334698 | 1.19E-31 |
| Adora2b | 0.59988362 | 8.52480906 | 3.82891298334698 | 1.19E-31 |
| 1700003E16Rik | 0.4316821 | 6.051072697 | 3.80914972863057 | 3.69E-25 |
| Cd40 | 0.131454 | 1.842646914 | 3.80914972863057 | 3.95E-07 |
| Rundc3b | 0.06020057 | 0.843857077 | 3.80914972863057 | 3.95E-07 |
| Zfp456 | 0.0813571 | 1.127309048 | 3.79247098740017 | 4.91E-10 |
| Gzme | 1.67785433 | 23.17162743 | 3.78767000066434 | 3.15E-42 |
| Rbm38 | 0.9353315 | 12.89996109 | 3.78574512355017 | 4.56E-45 |
| Sytl1 | 0.45971341 | 6.277344336 | 3.77134769519865 | 2.30E-24 |
| Ccl5 | 0.83328466 | 11.27773144 | 3.7585236553033 | 1.18E-12 |
| Serpina3f | 0.0981531 | 1.328410691 | 3.7585236553033 | 7.29E-07 |
| Grm4 | 0.05548961 | 0.751000175 | 3.7585236553033 | 7.29E-07 |
| Foxl1 | 0.03737113 | 0.505783395 | 3.7585236553033 | 0.000657 |
| Dydc2 | 0.18685564 | 2.528916973 | 3.7585236553033 | 0.000657 |
| Ppp1r3g | 0.19171324 | 2.501993619 | 3.7060562351328 | 4.00E-12 |
| Cck | 2.20200839 | 28.67516028 | 3.7029096805159 | 2.58E-47 |
| Pdxp | 0.88450606 | 11.22277997 | 3.66541425098288 | 3.65E-43 |
| Cda | 1.64081887 | 20.22419069 | 3.62359407565612 | 1.53E-31 |
| Eml5 | 0.04365373 | 0.538061374 | 3.62359407565612 | 2.47E-11 |
| Bcl3 | 1.32621682 | 16.25909532 | 3.61585842683876 | 2.97E-56 |
| Tnfsf15 | 0.34164957 | 4.146838657 | 3.60142269454874 | 1.06E-45 |
| Anks1b | 0.12652964 | 1.506988143 | 3.57412076327607 | 1.81E-21 |
| Hist1h4i | 7.96014301 | 93.13873019 | 3.54851495844394 | 2.65E-69 |
| Ankrd37 | 1.59075333 | 18.21715413 | 3.51751555628404 | 5.35E-31 |
| Mtmr7 | 0.2568045 | 2.917029511 | 3.50575758476457 | 2.20E-19 |
| Atp8b4 | 0.12004182 | 1.353878503 | 3.49548924925631 | 1.02E-14 |
| Mst1 | 0.14516869 | 1.637269082 | 3.49548924925631 | 6.29E-08 |
| Asb16 | 0.24365317 | 2.748015507 | 3.49548924925631 | 6.29E-08 |
| Flt4 | 0.13261342 | 1.483456398 | 3.48366381398059 | 8.56E-17 |
| Il1a | 0.22415104 | 2.491947714 | 3.47473068897828 | 4.98E-10 |
| Rab42 | 0.45150424 | 4.946748381 | 3.45366907333595 | 1.14E-07 |
| Adm2 | 0.76759202 | 8.244954556 | 3.42509992098116 | 3.67E-20 |
| P2ry2 | 0.58781024 | 6.286237039 | 3.41877428303359 | 1.35E-34 |
| Sfn | 1.16584945 | 12.46383595 | 3.41829474399052 | 1.18E-36 |
| Notch4 | 0.03350047 | 0.356240965 | 3.41060035120465 | 2.77E-05 |
| H2-T10 | 0.42545592 | 4.486869675 | 3.39862770947074 | 8.84E-24 |
| Gm14137 | 0.18033671 | 1.8828175 | 3.38412813996907 | 2.41E-11 |
| Rprm | 1.96991919 | 20.50847737 | 3.38001203215617 | 2.35E-53 |
| Tmem255a | 0.12896361 | 1.340219646 | 3.37743348846838 | 2.97E-09 |
| Il11 | 0.17070762 | 1.760280833 | 3.3662062329909 | 3.77E-07 |
| Cbs | 0.22097191 | 2.264348466 | 3.35716109249917 | 4.35E-11 |
| Lrrc3 | 0.48923469 | 5.003340125 | 3.35429291086967 | 1.82E-38 |
| Tslp | 2.05029949 | 20.67009379 | 3.33363837017032 | 1.48E-41 |
| Nanos1 | 0.76036165 | 7.650029029 | 3.33070755063929 | 7.35E-53 |
| Perm1 | 0.48946256 | 4.898730522 | 3.32313751122621 | 1.37E-33 |
| Esr2 | 0.0987078 | 0.986034765 | 3.32040254286327 | 6.81E-07 |
| Slc2a6 | 0.56966476 | 5.657250864 | 3.31191607693114 | 5.43E-22 |
| Rgs9 | 0.17399692 | 1.724113049 | 3.30872073775458 | 8.43E-24 |
| Slc15a3 | 0.51889293 | 5.1074417 | 3.29909203650611 | 9.74E-22 |
| Tnfaip3 | 0.82569821 | 8.054748061 | 3.28615298087784 | 1.29E-61 |
| Celf4 | 1.0490403 | 10.14127353 | 3.27309682798382 | 7.68E-70 |
| Slc22a4 | 1.32154892 | 12.77566653 | 3.27309682798382 | 4.52E-50 |
| Fgfbp3 | 1.44887608 | 13.88476641 | 3.26049679123561 | 2.28E-42 |
| Ptpn22 | 0.1610168 | 1.517665707 | 3.23657095206968 | 3.13E-08 |
| Ppp1r3e | 0.83620926 | 7.852829503 | 3.23127665241648 | 1.21E-25 |
| Trp53i11 | 1.53752289 | 14.38146795 | 3.22553114484348 | 5.08E-65 |
| Cpne5 | 0.07600907 | 0.710301135 | 3.22418722754919 | 2.21E-06 |
| Gdnf | 0.56828823 | 5.231006538 | 3.20239383674215 | 4.19E-40 |
| Sec14l5 | 0.18933426 | 1.738813993 | 3.19909624661074 | 5.61E-08 |
| Map3k9 | 0.04166816 | 0.38267335 | 3.19909624661074 | 5.61E-08 |
| Stmn4 | 0.15893468 | 1.459629419 | 3.19909624661074 | 0.000164 |
| Kcns3 | 0.0754303 | 0.692739226 | 3.19909624661074 | 0.000164 |
| Fam129c | 0.08913662 | 0.818615693 | 3.19909624661074 | 0.000164 |
| Ccno | 0.11793021 | 1.083051253 | 3.19909624661074 | 0.000164 |
| 1700012B09Rik | 0.32297384 | 2.966137447 | 3.19909624661074 | 0.000164 |
| Rai2 | 0.77763472 | 7.094681964 | 3.18957347252136 | 5.38E-28 |
| Rab11fip1 | 0.38886618 | 3.531004009 | 3.18273280045202 | 1.36E-47 |
| Avpr1a | 0.70404911 | 6.32574151 | 3.16748664013468 | 4.77E-29 |
| Hist1h4h | 11.4432971 | 102.5120788 | 3.16321921025332 | 3.99E-74 |
| Trim7 | 2.4293969 | 21.60660961 | 3.15280259425313 | 1.94E-49 |
| Apobec1 | 1.70933725 | 15.06089286 | 3.13929833004221 | 1.12E-56 |
| Gdf5 | 1.52906836 | 13.34981016 | 3.12609441564826 | 2.74E-51 |
| Sh2b2 | 1.11193679 | 9.674375325 | 3.12109373452219 | 2.13E-46 |
| Fcamr | 0.17365548 | 1.510884686 | 3.12109373452219 | 7.09E-06 |
| Ccbe1 | 0.71279797 | 6.183055402 | 3.11675479891872 | 1.59E-58 |
| Ddias | 1.28290564 | 11.06886942 | 3.10902090232473 | 1.15E-62 |
| Polr3g | 0.90374623 | 7.758178905 | 3.10172840975385 | 1.84E-39 |
| Tshr | 0.17957555 | 1.537592244 | 3.09801012147868 | 5.83E-12 |
| Nlrc5 | 0.71675079 | 6.112888849 | 3.09231083390861 | 3.72E-69 |
| Cadm2 | 0.17349206 | 1.47591928 | 3.08867225682604 | 6.00E-24 |
| Tnfrsf26 | 0.29726178 | 2.514475728 | 3.08045175014802 | 2.79E-13 |
| P2ry6 | 0.46163188 | 3.904848326 | 3.08045175014802 | 2.79E-13 |
| Aloxe3 | 0.1743397 | 1.474703357 | 3.08045175014802 | 3.19E-07 |
| Eva1c | 0.72299699 | 6.090719604 | 3.07455114872651 | 3.81E-22 |
| Slitrk6 | 0.7040784 | 5.873723507 | 3.06046737564684 | 7.93E-41 |
| Ppp1r14a | 1.66656933 | 13.89577623 | 3.05969318987247 | 4.95E-13 |
| Tbc1d2 | 1.51602644 | 12.61882155 | 3.05721037061195 | 4.49E-87 |
| Myh7b | 0.18039553 | 1.499769604 | 3.0555053928869 | 6.52E-16 |
| Igsf9b | 0.70457668 | 5.827426515 | 3.04803027245952 | 2.47E-40 |
| Slco4a1 | 0.28666936 | 2.355594467 | 3.03863157440825 | 8.75E-13 |
| Dpysl5 | 0.04406235 | 0.362065332 | 3.03863157440825 | 0.000528 |
| Rhbdl2 | 0.63626111 | 5.184294638 | 3.02645686049934 | 3.24E-11 |
| Rhbdf2 | 1.40458886 | 11.38925419 | 3.01945347270687 | 6.46E-70 |
| Nkd2 | 1.02996778 | 8.330602796 | 3.01582169155822 | 2.95E-43 |
| Fibcd1 | 0.06984964 | 0.56270817 | 3.01006242223957 | 2.25E-05 |
| Reep2 | 0.22973736 | 1.832254932 | 2.99556285246848 | 1.00E-06 |
| Tmem74 | 0.30059386 | 2.397366168 | 2.99556285246848 | 1.00E-06 |
| Gareml | 0.51960692 | 4.102230665 | 2.98091607660929 | 1.18E-25 |
| Rpgrip1 | 0.12731848 | 1.00516324 | 2.98091607660929 | 2.14E-09 |
| Scg5 | 1.17199756 | 9.238246473 | 2.97864946951293 | 7.25E-19 |
| Ppp1r3b | 1.4158334 | 11.15247847 | 2.97764094449835 | 1.01E-73 |
| Arhgef4 | 2.07868294 | 16.11542291 | 2.95470042411378 | 4.04E-68 |
| Ache | 0.45007543 | 3.480772316 | 2.95116873324569 | 4.04E-13 |
| Dusp9 | 0.12290949 | 0.95055165 | 2.95116873324569 | 3.98E-05 |
| Ccl6 | 0.23109723 | 1.787248923 | 2.95116873324569 | 3.98E-05 |
| Panx3 | 0.08786222 | 0.679504754 | 2.95116873324569 | 0.00094 |
| Wnt4 | 0.05787002 | 0.447552438 | 2.95116873324569 | 0.00094 |
| Casp4 | 2.06978555 | 15.56256599 | 2.91052674872703 | 3.31E-35 |
| Vnn1 | 2.28929096 | 17.17366702 | 2.90722538550425 | 1.13E-63 |
| 2610528A11Rik | 0.58528328 | 4.384985289 | 2.90536504358443 | 3.12E-06 |
| Prkaa2 | 0.6474442 | 4.837659978 | 2.90148160898042 | 7.84E-61 |
| Tmem132c | 0.36779096 | 2.739830811 | 2.89712889338471 | 2.12E-22 |
| Fam71f1 | 0.20792959 | 1.541072928 | 2.88976818845178 | 7.03E-05 |
| Il20ra | 0.31786937 | 2.30467803 | 2.85805932875472 | 2.03E-08 |
| Dgkh | 0.78077114 | 5.633941433 | 2.85117294319043 | 2.42E-34 |
| Map3k6 | 1.60834709 | 11.59945038 | 2.8504057562568 | 2.91E-75 |
| Reep1 | 1.63059705 | 11.73802429 | 2.84771738456476 | 6.77E-67 |
| Pde2a | 0.18349047 | 1.317707856 | 2.84425352927178 | 1.66E-09 |
| Plch2 | 0.15847928 | 1.129886564 | 2.83381178258224 | 1.38E-10 |
| Kank3 | 2.14181682 | 15.18392584 | 2.825637851094 | 1.09E-59 |
| Evpl | 0.29392235 | 2.072553222 | 2.81790220233409 | 1.02E-20 |
| D6Ertd527e | 0.34770084 | 2.448940319 | 2.81623915298401 | 2.90E-09 |
| Plekhn1 | 1.90203823 | 13.30664781 | 2.80652902803505 | 5.31E-44 |
| Tmc7 | 0.17234137 | 1.190040737 | 2.78767000087327 | 5.04E-09 |
| Vwa7 | 0.33342013 | 2.281059489 | 2.77429097117784 | 1.74E-15 |
| Rnf152 | 0.18157575 | 1.241267323 | 2.77317043122248 | 1.48E-16 |
| Celf5 | 1.7893006 | 12.22357486 | 2.77219859205959 | 2.32E-98 |
| Wfikkn1 | 0.39257368 | 2.65655947 | 2.7585236553033 | 6.00E-11 |
| Pomc | 0.4555953 | 3.08302893 | 2.7585236553033 | 1.32E-06 |
| Hist1h1d | 0.57019866 | 3.858553733 | 2.7585236553033 | 1.67E-05 |
| Fam89a | 0.25236169 | 1.707740131 | 2.7585236553033 | 0.000216 |
| Ticam2 | 0.10167145 | 0.688014177 | 2.7585236553033 | 0.000216 |
| Lcat | 0.24365317 | 1.648809304 | 2.7585236553033 | 0.000216 |
| Dpf1 | 0.77695199 | 5.210717175 | 2.74558459942102 | 3.20E-18 |
| Esm1 | 1.78841195 | 11.84799578 | 2.72789203656637 | 1.93E-36 |
| Gch1 | 0.80042358 | 5.300425941 | 2.72727272115546 | 5.09E-22 |
| St6gal1 | 0.73614822 | 4.839210373 | 2.71670347960867 | 5.88E-32 |
| Lhfpl1 | 0.31177717 | 2.049526528 | 2.71670347960867 | 2.30E-06 |
| Ccdc13 | 0.20950481 | 1.37721972 | 2.71670347960867 | 2.30E-06 |
| Stx11 | 2.67774427 | 17.58480879 | 2.71523955749874 | 3.02E-60 |
| Kcnb1 | 0.64468798 | 4.190038149 | 2.70029038970076 | 2.97E-66 |
| Tnfsf9 | 2.42821183 | 15.73626664 | 2.69612711798404 | 2.68E-28 |
| Ptger2 | 0.08807209 | 0.567606479 | 2.68813432726267 | 0.000376 |
| S100a7a | 0.44784834 | 2.886290434 | 2.68813432726267 | 0.000376 |
| Nqo1 | 6.6998342 | 43.13311851 | 2.68659872840055 | 1.14E-93 |
| Map7 | 0.76680374 | 4.886982486 | 2.67201466196478 | 1.35E-27 |
| Ret | 0.18082311 | 1.150800679 | 2.66998698068275 | 5.38E-13 |
| Fcna | 1.17589943 | 7.470166723 | 2.66737576708289 | 6.63E-15 |
| Krt80 | 0.49884347 | 3.134569983 | 2.65160845127199 | 6.38E-09 |
| Cntnap1 | 1.56847182 | 9.795913573 | 2.64282043391924 | 1.53E-72 |
| Rnf165 | 0.13637902 | 0.84963692 | 2.63922472675084 | 9.09E-10 |
| Mybl1 | 2.30825202 | 14.11809508 | 2.61267278868108 | 1.98E-95 |
| Twist2 | 5.22020072 | 31.80075884 | 2.60688400680604 | 4.20E-58 |
| Map3k8 | 3.04454695 | 18.42712811 | 2.59753177847554 | 7.80E-63 |
| Noct | 1.83823574 | 11.04563317 | 2.58708242140419 | 2.47E-46 |
| Tmem38a | 4.74984185 | 28.36689203 | 2.57825670961269 | 9.85E-80 |
| Abcb4 | 0.27145654 | 1.600775631 | 2.55997797579889 | 6.46E-10 |
| Unc13a | 0.20847821 | 1.222675275 | 2.55207277767188 | 4.03E-14 |
| Tspan12 | 2.66550697 | 15.58961968 | 2.54810387544792 | 5.89E-52 |
| Il6ra | 1.60506616 | 9.341555485 | 2.54103003329369 | 2.16E-42 |
| Pde1b | 0.34332259 | 1.991379154 | 2.53613123396685 | 1.10E-09 |
| Ccdc88b | 0.15614636 | 0.905698033 | 2.53613123396685 | 3.86E-07 |
| Oas1a | 0.23436131 | 1.359369414 | 2.53613123396685 | 0.000148 |
| Hspa4l | 2.26957655 | 13.04403718 | 2.52289540671044 | 4.03E-61 |
| Cd68 | 7.80628022 | 44.85010641 | 2.522404250784 | 2.69E-73 |
| Lamc2 | 0.66405397 | 3.810305799 | 2.52053437885095 | 8.90E-27 |
| Atf3 | 4.03063503 | 23.05424484 | 2.51595335192413 | 7.67E-60 |
| Panx1 | 3.30522864 | 18.81061548 | 2.50872507663847 | 2.42E-51 |
| Relt | 0.90316876 | 5.124779261 | 2.5044223741953 | 6.81E-20 |
| Cdk18 | 1.30737604 | 7.407655871 | 2.50234292033375 | 2.99E-30 |
| Mfsd9 | 1.43888328 | 8.142415781 | 2.50050732419882 | 3.58E-34 |
| Arntl | 2.62471774 | 14.78290865 | 2.49369596750167 | 5.27E-56 |
| Cd274 | 0.33447057 | 1.881245706 | 2.49173711463172 | 4.50E-10 |
| Jakmip3 | 0.19238007 | 1.074536647 | 2.48168344991578 | 2.21E-08 |
| Apln | 3.54907003 | 19.80441751 | 2.48030932369184 | 5.59E-80 |
| Car12 | 1.04188613 | 5.813047965 | 2.48009719886783 | 9.63E-29 |
| Dnah17 | 0.05943638 | 0.330385169 | 2.47473068923782 | 1.56E-07 |
| Dennd2c | 0.9340528 | 5.116811504 | 2.45366907359931 | 3.35E-33 |
| Gchfr | 1.05518479 | 5.780381653 | 2.45366907359931 | 7.97E-06 |
| Pm20d1 | 3.03107296 | 16.58430685 | 2.45191822986261 | 1.96E-74 |
| Serpini1 | 1.23576408 | 6.689966127 | 2.43659556041594 | 2.14E-27 |
| Arl5c | 0.85036673 | 4.589867431 | 2.43229542296004 | 3.09E-10 |
| Adrb2 | 0.87753799 | 4.71295992 | 2.42509992151841 | 1.36E-14 |
| Dffb | 1.78242763 | 9.555411176 | 2.42247445209789 | 1.63E-25 |
| 1810011O10Rik | 0.9142028 | 4.90094718 | 2.42247445209789 | 2.15E-09 |
| Arid3a | 0.63965206 | 3.410975072 | 2.41482493776217 | 7.46E-24 |
| Fam49a | 0.59325124 | 3.15429036 | 2.41060035174732 | 3.82E-20 |
| Alpl | 0.26038103 | 1.384434288 | 2.41060035174732 | 1.34E-05 |
| Cdh17 | 0.79880516 | 4.231766985 | 2.40534462632257 | 2.59E-19 |
| Ngf | 2.1272796 | 11.26594451 | 2.40488670049909 | 7.17E-18 |
| Tbx3 | 2.77877846 | 14.68509771 | 2.40183014207327 | 1.59E-86 |
| Ctxn1 | 4.0602237 | 21.36996725 | 2.3959535757054 | 3.08E-33 |
| Ccdc85a | 0.14181864 | 0.744250605 | 2.39174132455313 | 3.15E-06 |
| Fam19a2 | 0.78578706 | 4.116733772 | 2.38928984554875 | 3.45E-23 |
| Slc18a2 | 0.14543589 | 0.759216575 | 2.3841281405218 | 9.79E-05 |
| Tmem51 | 0.47222429 | 2.453731913 | 2.37743348791309 | 7.42E-07 |
| Cdk2ap2 | 9.10690497 | 46.95370388 | 2.36620623243127 | 1.66E-52 |
| Slc35g2 | 0.41874526 | 2.158981665 | 2.36620623243127 | 2.26E-05 |
| Gfpt2 | 4.07502991 | 20.91468298 | 2.35963357847328 | 1.21E-75 |
| Efna3 | 2.34782205 | 12.04678723 | 2.35925347168812 | 4.88E-19 |
| Fgf18 | 4.4592649 | 22.87535301 | 2.35891619651695 | 2.56E-43 |
| Filip1 | 0.18405747 | 0.940497086 | 2.35326717685737 | 5.28E-06 |
| Slc37a2 | 1.6208758 | 8.244471308 | 2.34665343495041 | 1.07E-44 |
| Slc2a9 | 1.47409491 | 7.481437887 | 2.34348615581126 | 2.69E-33 |
| Insl6 | 0.67041539 | 3.402542837 | 2.34348615581126 | 0.000725 |
| Fam131c | 0.30306449 | 1.538135803 | 2.34348615581126 | 0.000725 |
| Chd7 | 0.16632896 | 0.832342237 | 2.32313751064961 | 1.98E-12 |
| Proser2 | 0.91546377 | 4.572481211 | 2.32040254286327 | 8.06E-21 |
| Galr2 | 0.68423839 | 3.417576163 | 2.32040254286327 | 4.00E-09 |
| Acat3 | 0.33335572 | 1.665017016 | 2.32040254286327 | 3.79E-05 |
| Pde4b | 3.7454916 | 18.49563436 | 2.30395779186151 | 1.08E-94 |
| Hmgcll1 | 0.42839822 | 2.100285284 | 2.29356093062972 | 3.84E-10 |
| Vwa1 | 1.76144171 | 8.548153569 | 2.27885609739201 | 4.03E-32 |
| Thsd1 | 0.44946585 | 2.17253622 | 2.27309682813306 | 2.17E-12 |
| Cgn | 0.15369785 | 0.742913283 | 2.27309682813306 | 1.47E-05 |
| Wdr72 | 0.14839589 | 0.717285765 | 2.27309682813306 | 1.47E-05 |
| Pnp2 | 0.54269112 | 2.623149483 | 2.27309682813306 | 6.31E-05 |
| Bcam | 2.2059368 | 10.61817926 | 2.26707304032421 | 1.78E-30 |
| Fah | 6.84382375 | 32.60770219 | 2.25233826772835 | 1.69E-59 |
| Mlkl | 5.17672966 | 24.65347013 | 2.25167784441843 | 1.11E-57 |
| Aif1l | 2.99677722 | 14.26793155 | 2.25129245770108 | 8.21E-49 |
| Lrrc15 | 0.22923963 | 1.087905289 | 2.24662461663368 | 7.57E-08 |
| Arhgap27 | 1.43156258 | 6.778857317 | 2.24345136624511 | 4.41E-36 |
| Dcbld1 | 2.04781428 | 9.650848774 | 2.23657095191661 | 4.77E-34 |
| Itprip | 4.40594287 | 20.72863894 | 2.23410269631276 | 3.56E-88 |
| Serpinb2 | 0.8333893 | 3.920846521 | 2.23410269631276 | 4.15E-10 |
| Sacs | 0.19853761 | 0.927662207 | 2.22418722770357 | 0.000105 |
| B3gnt8 | 0.35549611 | 1.661047004 | 2.22418722770357 | 0.000105 |
| Wnt11 | 2.01324277 | 9.37733986 | 2.21965756919975 | 8.42E-33 |
| H2-Q10 | 1.20155915 | 5.590056799 | 2.21795527380493 | 1.64E-10 |
| Spire2 | 0.23707359 | 1.100081206 | 2.21420313907949 | 0.000457 |
| Tmem154 | 2.41626372 | 11.19927325 | 2.21255528596776 | 1.18E-42 |
| Ptgir | 0.43132604 | 1.988631601 | 2.20492532541927 | 1.17E-08 |
| Ankrd13d | 2.11833727 | 9.739713599 | 2.20094704230069 | 2.69E-24 |
| Kbtbd11 | 0.88850232 | 4.079928014 | 2.19909624661074 | 1.47E-32 |
| Adssl1 | 7.04104604 | 32.18262181 | 2.19242018158704 | 4.43E-64 |
| Pbx4 | 0.57485507 | 2.619838901 | 2.18820793050132 | 4.01E-05 |
| Dusp4 | 0.77483114 | 3.52491341 | 2.18563398664021 | 1.06E-10 |
| Itgb7 | 0.4136819 | 1.879596802 | 2.18382948987721 | 8.59E-07 |
| Il7 | 0.43223355 | 1.960673488 | 2.18146635256963 | 1.92E-08 |
| Klhdc7a | 1.43611359 | 6.503176216 | 2.17897465081881 | 1.44E-42 |
| Gimap6 | 0.41173154 | 1.857467554 | 2.17356115436895 | 0.000173 |
| Kif17 | 0.3108056 | 1.393049831 | 2.16416245663842 | 3.35E-07 |
| Filip1l | 3.56020133 | 15.90776852 | 2.15970073952511 | 2.29E-68 |
| Abcc3 | 0.7519053 | 3.35648111 | 2.15832664095352 | 2.10E-19 |
| Trim72 | 0.223743 | 0.99496456 | 2.15280259441535 | 0.000756 |
| Gpr75 | 0.18928566 | 0.841735932 | 2.15280259441535 | 0.000756 |
| Dgka | 5.26942205 | 23.37941424 | 2.14952214297327 | 3.95E-71 |
| Slc4a8 | 0.15946029 | 0.707291675 | 2.14910811070454 | 2.83E-10 |
| Wdr91 | 5.62111651 | 24.8992625 | 2.1471743881338 | 6.38E-71 |
| Mapk13 | 11.2371859 | 49.77041869 | 2.14700773553159 | 1.61E-74 |
| Sema4g | 1.6452676 | 7.281561411 | 2.14592558751005 | 1.12E-34 |
| Piga | 1.01597879 | 4.49415423 | 2.14517935317279 | 1.38E-18 |
| Nr4a3 | 0.91371032 | 4.032462331 | 2.14185229488323 | 3.14E-25 |
| H2-T24 | 0.45626345 | 2.00935815 | 2.13879573634867 | 5.93E-06 |
| Gfod1 | 0.61510159 | 2.69994382 | 2.13403279020008 | 2.16E-20 |
| Mllt11 | 3.16807272 | 13.86693785 | 2.12997188591544 | 4.66E-38 |
| Nxpe3 | 0.55051217 | 2.394857967 | 2.12109373452219 | 1.47E-17 |
| Foxf1 | 1.00354963 | 4.365677937 | 2.12109373452219 | 1.67E-12 |
| Slc22a23 | 0.31853043 | 1.385682596 | 2.12109373452219 | 1.81E-10 |
| 2700097O09Rik | 1.00107275 | 4.35490292 | 2.12109373452219 | 2.29E-06 |
| C77080 | 2.96432946 | 12.88522515 | 2.11994003991643 | 4.97E-71 |
| Whamm | 4.61361127 | 20.03545449 | 2.11858687904369 | 1.96E-65 |
| Cd80 | 8.39399502 | 36.42150475 | 2.11736101578075 | 7.89E-66 |
| Ugt1a7c | 1.74625502 | 7.564157595 | 2.11491515958595 | 2.07E-27 |
| Map3k13 | 0.24732122 | 1.067936065 | 2.11036732787221 | 7.82E-09 |
| Homez | 0.7485898 | 3.228708753 | 2.10871001005206 | 8.96E-21 |
| Mgat5 | 3.56374159 | 15.33086081 | 2.10497406907321 | 1.29E-50 |
| Gcnt2 | 2.53421624 | 10.85294796 | 2.09847543180702 | 3.33E-50 |
| Rab26 | 1.8513563 | 7.904692066 | 2.0941266871159 | 4.23E-13 |
| Wdr54 | 3.05576075 | 12.99787477 | 2.08867225699563 | 2.40E-18 |
| Pgbd1 | 0.50495377 | 2.147853317 | 2.08867225699563 | 1.26E-08 |
| Fbxo34 | 6.46334376 | 27.48541947 | 2.08831386785055 | 2.78E-89 |
| Dyrk3 | 0.96097186 | 4.077233488 | 2.08502447996159 | 4.72E-10 |
| Amz1 | 0.82397422 | 3.464999729 | 2.07218413412129 | 7.30E-11 |
| Lamc3 | 0.11192432 | 0.468864009 | 2.06664595055083 | 0.000464 |
| Slc9a3r1 | 11.3381125 | 47.40394153 | 2.06382653391882 | 5.39E-93 |
| Pitpnm2 | 2.97507073 | 12.36551082 | 2.05532596440334 | 2.13E-87 |
| Snapc1 | 9.73443144 | 40.40962807 | 2.0535304507936 | 3.40E-87 |
| Pvrl1 | 4.43698151 | 18.38277864 | 2.05070440684635 | 3.72E-94 |
| Ppfia3 | 0.25006246 | 1.032889817 | 2.04632596606377 | 2.32E-06 |
| Plaur | 10.6973516 | 44.12302786 | 2.044278137404 | 1.11E-62 |
| Nova2 | 0.19819375 | 0.817483502 | 2.044278137404 | 3.28E-08 |
| Hip1r | 1.83860504 | 7.572273644 | 2.04211494454427 | 1.49E-34 |
| Cdh23 | 0.13963209 | 0.568865438 | 2.02645686032228 | 1.35E-07 |
| Smim3 | 9.91445113 | 40.38408633 | 2.02618208745958 | 3.53E-80 |
| Crem | 2.92491179 | 11.89934975 | 2.02441771529111 | 2.42E-33 |
| Pkn3 | 1.4341537 | 5.801089264 | 2.01612417496445 | 4.04E-18 |
| Fez1 | 3.52946311 | 14.23053491 | 2.0114692435746 | 2.52E-19 |
| Ubash3b | 0.40931929 | 1.648736673 | 2.01006242223957 | 1.44E-06 |
| Lancl3 | 0.70547538 | 2.837105689 | 2.00775226168381 | 2.83E-12 |
| Stk10 | 2.46400452 | 9.907435142 | 2.00750671744631 | 2.71E-50 |
| Unc5b | 4.18566826 | 16.80517639 | 2.00537580739615 | 7.27E-97 |
| Gstm6 | 2.97671416 | 0.742618476 | -2.00302757695464 | 0.00012 |
| Zfp641 | 0.93361686 | 0.232914916 | -2.00302757695464 | 0.00012 |
| Galnt13 | 3.01715797 | 0.75221303 | -2.00397703142689 | 4.74E-22 |
| D3Ertd751e | 3.00223791 | 0.748020849 | -2.0048879214351 | 5.87E-12 |
| Mtus1 | 5.77580769 | 1.436711654 | -2.00725216439352 | 1.57E-38 |
| Plekhf1 | 49.6683626 | 12.34680623 | -2.00818927976969 | 3.82E-77 |
| Actg2 | 2.94421967 | 0.731888628 | -2.00818927976969 | 4.10E-05 |
| Dock3 | 0.42719286 | 0.106193705 | -2.00818927976969 | 4.10E-05 |
| Dnah7b | 1.43683766 | 0.355935987 | -2.01320735975351 | 6.60E-19 |
| Tox | 4.69314279 | 1.162359354 | -2.01349819147569 | 1.20E-14 |
| P4ha3 | 21.2241416 | 5.25400213 | -2.01421751104257 | 1.01E-50 |
| Mybl2 | 1.28487228 | 0.317749235 | -2.01566440254777 | 4.89E-06 |
| Snx32 | 18.905714 | 4.666344462 | -2.01845761721333 | 2.83E-32 |
| Sorl1 | 11.3042007 | 2.789796478 | -2.01862719893891 | 1.23E-78 |
| Thy1 | 20.2747524 | 4.992450667 | -2.02186421777222 | 2.88E-36 |
| Runx1t1 | 12.6555732 | 3.115500368 | -2.02223705623151 | 3.22E-95 |
| Idi1 | 17.5168527 | 4.289418196 | -2.02988970173825 | 2.47E-51 |
| Pdzd7 | 2.4657476 | 0.602771031 | -2.03234314606861 | 4.94E-11 |
| Mthfd2 | 21.5893187 | 5.269362406 | -2.03461740721272 | 5.35E-46 |
| Osr2 | 22.3177754 | 5.440657469 | -2.03634032334302 | 1.46E-39 |
| Farsb | 13.1341273 | 3.197766104 | -2.03818402496891 | 2.42E-31 |
| Adam33 | 7.93377831 | 1.925880712 | -2.04248974239393 | 1.15E-26 |
| Thbs4 | 22.0753424 | 5.343513334 | -2.04657529508979 | 2.65E-72 |
| Il16 | 2.74938842 | 0.664471613 | -2.04883126526194 | 2.75E-15 |
| Drp2 | 1.01383166 | 0.245022622 | -2.04883126526194 | 1.56E-08 |
| Scube3 | 2.07734346 | 0.502051928 | -2.04883126526194 | 1.26E-07 |
| Plcl1 | 0.74251956 | 0.17945197 | -2.04883126526194 | 2.97E-06 |
| Fam109b | 1.61192771 | 0.389570348 | -2.04883126526194 | 8.58E-06 |
| Ptpn6 | 1.3945606 | 0.337037111 | -2.04883126526194 | 0.000212 |
| Astn2 | 0.64113415 | 0.154949167 | -2.04883126526194 | 0.000212 |
| Chek1 | 0.91178072 | 0.220358971 | -2.04883126526194 | 0.000212 |
| Casp12 | 14.4802003 | 3.489309079 | -2.05306826183543 | 1.60E-39 |
| Fibin | 43.4668305 | 10.44044767 | -2.05773132719312 | 3.83E-92 |
| Ndrg3 | 35.2511558 | 8.449741649 | -2.06069142083806 | 5.48E-97 |
| Adhfe1 | 3.27732531 | 0.783546478 | -2.06442812388165 | 6.53E-12 |
| Nuak2 | 10.7369208 | 2.560753367 | -2.06794008685657 | 9.99E-36 |
| Cd59a | 6.28043125 | 1.489214556 | -2.07631200156925 | 1.79E-13 |
| Msx1 | 3.03613805 | 0.719928424 | -2.07631200156925 | 2.19E-07 |
| Birc5 | 1.71627124 | 0.406961882 | -2.07631200156925 | 2.19E-07 |
| Sfrp1 | 16.6231428 | 3.937984162 | -2.07766395184478 | 1.01E-75 |
| Zfp128 | 1.41411647 | 0.334788606 | -2.07857861114327 | 6.27E-07 |
| Ctf1 | 3.58633592 | 0.84748391 | -2.08125274308999 | 1.80E-06 |
| Kif15 | 1.03210331 | 0.243895432 | -2.08125274308999 | 1.80E-06 |
| Sort1 | 9.14638137 | 2.160344658 | -2.08193957967486 | 1.95E-66 |
| St3gal6 | 5.9756246 | 1.41060193 | -2.08277859590611 | 3.16E-11 |
| Slc7a5 | 26.0723457 | 6.148957085 | -2.0841067396253 | 2.43E-95 |
| Cep128 | 1.88698153 | 0.444922241 | -2.08445517499266 | 8.92E-11 |
| Tk1 | 5.97938049 | 1.406038781 | -2.08835962828785 | 7.13E-10 |
| Mrgprf | 25.9176039 | 6.077180911 | -2.0924582011182 | 3.79E-55 |
| Tubb2a | 45.8443976 | 10.71366942 | -2.09729275759262 | 1.19E-77 |
| Dchs1 | 5.8471728 | 1.365453032 | -2.09835954048113 | 5.92E-66 |
| Mdga2 | 0.32620883 | 0.076119527 | -2.09945733939788 | 0.000128 |
| Tbxa2r | 1.64594029 | 0.384073589 | -2.09945733939788 | 0.000128 |
| Wdr62 | 2.42502699 | 0.563538473 | -2.10541479673242 | 1.85E-13 |
| Bmf | 1.79071669 | 0.41546883 | -2.10772495705151 | 4.32E-10 |
| Dach2 | 0.46160298 | 0.107097707 | -2.10772495705151 | 0.000377 |
| Cplx2 | 6.46865772 | 1.498204504 | -2.11023180862838 | 8.78E-35 |
| Bdh2 | 8.69078189 | 2.009065186 | -2.11296160322434 | 4.12E-12 |
| Plxna2 | 8.15980886 | 1.879999072 | -2.11780340814109 | 1.12E-95 |
| Kng2 | 2.83145067 | 0.649509262 | -2.12411939363215 | 2.80E-08 |
| Oasl2 | 2.68080643 | 0.613796904 | -2.12683377792649 | 2.61E-10 |
| Prdm16 | 2.60959482 | 0.596511204 | -2.12920468073106 | 3.05E-25 |
| Id4 | 9.06272368 | 2.061437313 | -2.13629410539301 | 1.84E-17 |
| Tsga10 | 1.04270317 | 0.237176735 | -2.13629410539301 | 2.62E-05 |
| Casc5 | 0.57640312 | 0.131110572 | -2.13629410539301 | 2.62E-05 |
| Anln | 4.77490097 | 1.084954498 | -2.13783627414261 | 5.27E-29 |
| Prr5l | 2.85816058 | 0.647198225 | -2.14280741490592 | 1.46E-14 |
| Mrc1 | 1.60165993 | 0.361953195 | -2.14569280584108 | 1.57E-10 |
| Herpud1 | 47.7120048 | 10.74514559 | -2.1506672763223 | 1.03E-96 |
| Cep112 | 2.72897142 | 0.61352301 | -2.15316792746446 | 1.19E-11 |
| Pdia5 | 42.9877462 | 9.635567096 | -2.15748399225497 | 8.17E-85 |
| Gins1 | 3.96883443 | 0.885403446 | -2.16430848268188 | 5.40E-06 |
| Usp11 | 10.6978091 | 2.377065146 | -2.17006201276123 | 1.36E-41 |
| Gpx7 | 38.0877672 | 8.456307921 | -2.17122789886021 | 1.59E-51 |
| Mef2c | 5.92076823 | 1.314458968 | -2.17131527352488 | 1.07E-42 |
| Sox6 | 8.18267162 | 1.805622319 | -2.18007579967723 | 7.48E-79 |
| Mcm6 | 9.18700573 | 2.027242855 | -2.18007579967723 | 8.75E-46 |
| Nox4 | 2.63377474 | 0.581179677 | -2.18007579967723 | 1.53E-12 |
| Hrasls | 0.8348569 | 0.18342549 | -2.18633478960882 | 0.000665 |
| Cadm3 | 0.82451905 | 0.179985435 | -2.19567265572316 | 4.54E-05 |
| Ercc6l | 0.81685915 | 0.178313345 | -2.19567265572316 | 4.54E-05 |
| Mettl24 | 2.24275651 | 0.489574509 | -2.19567265572316 | 4.54E-05 |
| Atp6v0a4 | 3.50233012 | 0.760847105 | -2.20263659984862 | 2.58E-12 |
| Tagln | 36.0591328 | 7.821373914 | -2.20487074466909 | 1.57E-64 |
| Sv2c | 1.50890764 | 0.326947739 | -2.20637254569234 | 1.72E-08 |
| Kif4 | 1.36160341 | 0.295030091 | -2.20637254569234 | 1.72E-08 |
| Dhrs9 | 12.133876 | 2.619331623 | -2.211769837037 | 2.22E-39 |
| Mtap7d3 | 3.91815219 | 0.841722838 | -2.21875626745044 | 1.17E-13 |
| Foxm1 | 2.28079462 | 0.489975077 | -2.21875626745044 | 1.54E-12 |
| Itga8 | 1.72183821 | 0.369896439 | -2.21875626745044 | 1.54E-12 |
| Klf12 | 1.56344472 | 0.335869323 | -2.21875626745044 | 2.03E-11 |
| Pcdhb20 | 2.34457899 | 0.503677648 | -2.21875626745044 | 2.70E-10 |
| Rps6ka6 | 0.96847322 | 0.205516452 | -2.23645826843772 | 1.91E-06 |
| Lrp4 | 7.67495344 | 1.625102316 | -2.23962744543053 | 1.01E-70 |
| Fam174b | 1.3218048 | 0.279521816 | -2.24147634576267 | 2.69E-05 |
| Bcl11a | 1.41168947 | 0.297436196 | -2.24677064743872 | 3.40E-11 |
| Syne3 | 7.85204964 | 1.647599613 | -2.25270360193211 | 5.34E-50 |
| Socs2 | 19.3159285 | 4.042625153 | -2.25642668594948 | 3.19E-50 |
| Nusap1 | 1.38273174 | 0.289019083 | -2.25828463126398 | 5.49E-06 |
| Cep41 | 4.01140626 | 0.837627106 | -2.25972805256928 | 3.13E-17 |
| Nlgn3 | 0.79766137 | 0.165238827 | -2.27122368560348 | 7.82E-05 |
| Fancd2 | 0.64797471 | 0.13423062 | -2.27122368560348 | 7.82E-05 |
| Nrxn2 | 0.46506293 | 0.096339694 | -2.27122368560348 | 7.82E-05 |
| Hrct1 | 3.39246341 | 0.70276272 | -2.27122368560348 | 7.82E-05 |
| Rad51c | 0.99209452 | 0.205516452 | -2.27122368560348 | 7.82E-05 |
| Chdh | 3.77731388 | 0.775728854 | -2.28373630005949 | 3.95E-26 |
| Stk26 | 6.77053164 | 1.379626044 | -2.29499185155957 | 1.09E-27 |
| Mmp16 | 12.4010731 | 2.523864192 | -2.29675878094407 | 2.84E-67 |
| Dync1i1 | 1.52799145 | 0.310976548 | -2.29675878094407 | 3.23E-06 |
| Mis18bp1 | 1.0464288 | 0.212969003 | -2.29675878094407 | 3.23E-06 |
| Dner | 1.15735256 | 0.235544186 | -2.29675878094407 | 3.23E-06 |
| Nek2 | 2.14524536 | 0.434839471 | -2.30258785658935 | 2.12E-09 |
| Htra4 | 16.4837346 | 3.334122017 | -2.30566433649229 | 2.01E-43 |
| Slc24a3 | 19.3630932 | 3.906233949 | -2.30945917280364 | 8.89E-79 |
| Dock8 | 1.96875505 | 0.397077434 | -2.30979134347541 | 1.78E-19 |
| Myom1 | 2.46344511 | 0.495343591 | -2.31417583499726 | 1.08E-17 |
| Slc8a3 | 2.36212029 | 0.473935401 | -2.31732009996682 | 3.07E-15 |
| Inpp4b | 0.38340357 | 0.076684877 | -2.32184975867345 | 4.58E-05 |
| Arsj | 15.6784542 | 3.129865188 | -2.32461089876505 | 1.54E-69 |
| Deptor | 4.52877397 | 0.900627952 | -2.33011738046068 | 3.21E-47 |
| Cd1d1 | 13.4446841 | 2.669074762 | -2.33262422996483 | 7.94E-31 |
| Adcy5 | 0.96190034 | 0.190204258 | -2.33833788378347 | 3.90E-07 |
| Sema3d | 2.40880732 | 0.47556736 | -2.3405973898449 | 3.67E-20 |
| Slc12a5 | 0.89993499 | 0.177547834 | -2.34161301941458 | 8.08E-08 |
| Chtf18 | 1.9231813 | 0.378721077 | -2.34428715258685 | 1.68E-08 |
| Pmf1 | 7.59348042 | 1.487992494 | -2.35139404025692 | 3.26E-11 |
| Dact1 | 6.85299644 | 1.336357843 | -2.35842855750828 | 2.08E-32 |
| Adamtsl1 | 1.83446997 | 0.354683451 | -2.37075936164167 | 7.66E-19 |
| Hcn1 | 0.69914384 | 0.135175149 | -2.37075936164167 | 4.73E-08 |
| Rarres1 | 2.74390423 | 0.530516955 | -2.37075936164167 | 5.41E-06 |
| B3galt2 | 0.72489213 | 0.140153421 | -2.37075936164167 | 2.67E-05 |
| Dus4l | 1.56417617 | 0.302423814 | -2.37075936164167 | 0.000133 |
| Cnr1 | 0.47622929 | 0.092075995 | -2.37075936164167 | 0.000133 |
| Kcnt2 | 0.69887882 | 0.135123908 | -2.37075936164167 | 0.000133 |
| Degs2 | 0.69462191 | 0.134300861 | -2.37075936164167 | 0.000676 |
| Ptchd1 | 1.29557851 | 0.247196164 | -2.38986818628072 | 1.12E-11 |
| D17H6S56E-5 | 11.1749336 | 2.122022249 | -2.39675456705422 | 6.69E-47 |
| Apcdd1 | 4.42632239 | 0.840519947 | -2.39675456705422 | 1.27E-16 |
| Ddx60 | 0.75665 | 0.142725473 | -2.40638326988003 | 6.45E-07 |
| Spc24 | 3.36201638 | 0.634170856 | -2.40638326988003 | 6.45E-07 |
| AA414768 | 10.2068634 | 1.922176044 | -2.40872721422847 | 1.91E-22 |
| Asb13 | 5.18667839 | 0.976188935 | -2.40957861005735 | 7.43E-17 |
| Zbtb8a | 3.57314258 | 0.671654934 | -2.41140134635221 | 3.10E-11 |
| Egr2 | 2.49964321 | 0.468864009 | -2.41448074182612 | 1.47E-10 |
| Bambi | 3.62144027 | 0.678965464 | -2.4151534812333 | 5.13E-24 |
| Hoxc11 | 3.74773192 | 0.701226627 | -2.41806507816111 | 1.55E-05 |
| Tspyl4 | 4.73674257 | 0.877432292 | -2.43253555582391 | 1.76E-24 |
| Aqp5 | 11.5539673 | 2.137362268 | -2.43448495872251 | 8.17E-24 |
| Pycr1 | 4.783094 | 0.880744494 | -2.44114868878688 | 6.75E-19 |
| Pparg | 5.46071772 | 1.005520081 | -2.44114868878688 | 5.58E-16 |
| Cmklr1 | 1.63303291 | 0.300701752 | -2.44114868878688 | 3.74E-07 |
| Tnfsfm13 | 1.03936882 | 0.191386238 | -2.44114868878688 | 0.00039 |
| Mnd1 | 2.65788252 | 0.489414465 | -2.44114868878688 | 0.00039 |
| Adam22 | 2.15025238 | 0.392641475 | -2.45322152227257 | 1.61E-26 |
| Cdc20 | 8.55681524 | 1.558499762 | -2.45691600516179 | 1.05E-20 |
| E2f7 | 0.64641951 | 0.117169898 | -2.46386876255097 | 8.95E-06 |
| Hlf | 1.77692622 | 0.320905566 | -2.46916306649274 | 3.37E-14 |
| Lmod1 | 2.10461452 | 0.379786618 | -2.47029503732453 | 6.10E-12 |
| Rasl10b | 1.36644561 | 0.245761702 | -2.47509601753895 | 2.17E-07 |
| Cenph | 3.83906148 | 0.690473353 | -2.47509601753895 | 2.17E-07 |
| Camsap3 | 1.10721536 | 0.199137915 | -2.47509601753895 | 2.17E-07 |
| Kif14 | 0.35820347 | 0.064126326 | -2.4817906719411 | 4.44E-05 |
| Ung | 1.54431259 | 0.27646603 | -2.4817906719411 | 4.44E-05 |
| A230050P20Rik | 7.82151286 | 1.397870073 | -2.48421741400267 | 2.90E-18 |
| Dmd | 0.73468854 | 0.131239534 | -2.48493038428235 | 1.95E-14 |
| Sntg2 | 3.59735086 | 0.640615682 | -2.48940386091182 | 3.54E-12 |
| Prr11 | 1.0983811 | 0.195599536 | -2.48940386091182 | 1.05E-06 |
| Klhl23 | 1.17398926 | 0.208454309 | -2.49361611042211 | 2.59E-08 |
| P2rx5 | 6.91365869 | 1.21519387 | -2.50826288613778 | 6.93E-22 |
| Ube2ql1 | 2.72215644 | 0.478465595 | -2.50826288613778 | 7.93E-11 |
| Phf19 | 1.30035151 | 0.228559039 | -2.50826288613778 | 1.25E-07 |
| Lrrc4c | 0.63046836 | 0.110815607 | -2.50826288613778 | 0.000223 |
| Asphd2 | 1.53443119 | 0.269702549 | -2.50826288613778 | 0.000223 |
| Cldn1 | 0.75088178 | 0.131980328 | -2.50826288613778 | 0.000223 |
| Iigp1 | 6.2694465 | 1.095736772 | -2.51643681287322 | 7.76E-27 |
| Top2a | 7.65445517 | 1.332357473 | -2.52231847852372 | 2.91E-53 |
| Pbk | 2.62097389 | 0.454774863 | -2.52687856760965 | 6.05E-07 |
| Acot1 | 2.76901349 | 0.480461761 | -2.52687856760965 | 6.05E-07 |
| Igsf9 | 0.73728138 | 0.127275721 | -2.53425809004441 | 2.54E-05 |
| Gbp9 | 0.85255136 | 0.147174595 | -2.53425809004441 | 2.54E-05 |
| Pola1 | 1.86191669 | 0.319991204 | -2.54068436401671 | 1.77E-14 |
| Draxin | 0.95727582 | 0.164518555 | -2.54068436401671 | 7.17E-08 |
| Il17d | 7.15128035 | 1.225138174 | -2.54525708956929 | 6.80E-13 |
| Frzb | 10.7966725 | 1.831114051 | -2.55979318394238 | 4.42E-43 |
| Mfi2 | 1.52595758 | 0.25880218 | -2.55979318394238 | 1.03E-09 |
| Rnf157 | 4.34869874 | 0.735120502 | -2.56453110219353 | 2.88E-28 |
| Slfn5 | 5.3009719 | 0.892778933 | -2.56988200583412 | 1.47E-43 |
| Gcat | 3.21646827 | 0.540768954 | -2.57239322654222 | 1.51E-11 |
| Ptprd | 7.53859223 | 1.265388687 | -2.57471453380944 | 1.16E-92 |
| Tubb2b | 5.98560253 | 1.001491722 | -2.57934598196072 | 8.88E-17 |
| Dlgap5 | 2.16460029 | 0.360786304 | -2.58488417027771 | 5.91E-10 |
| Kcnk6 | 0.78452865 | 0.130761874 | -2.58488417027771 | 1.45E-05 |
| Scin | 1.07038293 | 0.178406841 | -2.58488417027771 | 1.45E-05 |
| Cml1 | 3.8295241 | 0.634641309 | -2.59315178049085 | 1.68E-06 |
| Itga10 | 13.7148556 | 2.269840405 | -2.59507666283154 | 6.39E-94 |
| Shroom3 | 2.86902452 | 0.474616635 | -2.59572572482313 | 3.16E-29 |
| Cygb | 1.94567143 | 0.321132314 | -2.59902834782242 | 1.98E-07 |
| Nr3c2 | 1.10970396 | 0.183156208 | -2.59902834782242 | 1.98E-07 |
| Gfra1 | 3.3441711 | 0.55027647 | -2.60342012085703 | 1.82E-22 |
| Kif2c | 1.83712768 | 0.302295578 | -2.60342012085703 | 2.35E-08 |
| Pgr | 1.60572709 | 0.263888813 | -2.6052246183512 | 2.37E-16 |
| Gcnt1 | 3.674476 | 0.603639508 | -2.60577939210944 | 2.80E-24 |
| Gxylt2 | 15.6901433 | 2.576796641 | -2.60620793719417 | 4.56E-40 |
| Il17rb | 3.48869725 | 0.570746078 | -2.6117674631805 | 4.08E-11 |
| Camk4 | 3.57036563 | 0.581038282 | -2.61936669891167 | 4.81E-61 |
| Zfp354c | 7.23441535 | 1.175570444 | -2.62151543751997 | 4.46E-54 |
| Prc1 | 6.10687472 | 0.989830643 | -2.62518063487891 | 1.42E-26 |
| Sec16b | 8.78689157 | 1.419621479 | -2.62984658687319 | 2.31E-56 |
| Galnt15 | 1.13406448 | 0.182720309 | -2.63379376896782 | 7.20E-05 |
| Iqgap2 | 3.58441636 | 0.574432313 | -2.64152941302964 | 8.97E-30 |
| Gpc3 | 25.4598225 | 4.054932633 | -2.65047250647031 | 1.42E-80 |
| Malrd1 | 2.49466142 | 0.396650818 | -2.65290259060225 | 1.38E-24 |
| Sugct | 4.3014754 | 0.682709035 | -2.65548883993147 | 1.33E-11 |
| Gdf7 | 4.74190505 | 0.751490236 | -2.65764050942983 | 1.10E-10 |
| Galnt5 | 1.4649206 | 0.230538782 | -2.66774109662727 | 6.39E-08 |
| Lpar4 | 13.6804613 | 2.145303316 | -2.6728633331197 | 1.29E-81 |
| Ndc80 | 1.85955745 | 0.291514023 | -2.67332213514428 | 5.42E-07 |
| Pcdh17 | 2.01251522 | 0.314884604 | -2.67610457084106 | 4.93E-28 |
| Ecm2 | 5.13717909 | 0.802920088 | -2.67764805971798 | 3.96E-27 |
| C1qtnf3 | 4.0438381 | 0.630525121 | -2.68109948553702 | 9.53E-16 |
| Nell2 | 1.95537822 | 0.303798468 | -2.68626119184668 | 5.17E-10 |
| Podxl2 | 27.5993255 | 4.277482424 | -2.68979917599166 | 2.46E-78 |
| 2810408A11Rik | 1.65200925 | 0.255524637 | -2.69268745466358 | 4.06E-05 |
| Lrp3 | 8.17037281 | 1.258398059 | -2.69881355916711 | 3.79E-47 |
| Gm1673 | 12.624942 | 1.93726559 | -2.70418309462068 | 3.53E-11 |
| Fam84b | 3.14178126 | 0.480572805 | -2.70875582151532 | 4.72E-26 |
| Knstrn | 3.37035318 | 0.514450259 | -2.7117962754971 | 3.07E-16 |
| Epha5 | 0.80971577 | 0.123594908 | -2.7117962754971 | 2.93E-10 |
| Rad51ap1 | 2.29073814 | 0.349657964 | -2.7117962754971 | 3.06E-07 |
| Gucy1b3 | 0.64649408 | 0.09868077 | -2.7117962754971 | 0.000363 |
| Neil3 | 0.93286828 | 0.14239289 | -2.7117962754971 | 0.000363 |
| Ednra | 10.2025334 | 1.555768381 | -2.71322823629328 | 8.83E-54 |
| Ccdc102a | 24.9659897 | 3.805919691 | -2.71364707935057 | 2.45E-82 |
| Ak5 | 9.5182565 | 1.447423959 | -2.71720977267838 | 3.96E-43 |
| Fxyd6 | 13.3952137 | 2.028939766 | -2.72291966150378 | 2.03E-35 |
| D630003M21Rik | 2.20112061 | 0.331001293 | -2.73332944315832 | 1.42E-15 |
| Ube2c | 5.34676075 | 0.80403805 | -2.73332944315832 | 2.04E-08 |
| Mapk12 | 3.80434129 | 0.565804553 | -2.74927098340304 | 1.13E-11 |
| Mxd3 | 2.21407392 | 0.32929041 | -2.74927098340304 | 2.28E-05 |
| Cenpm | 2.38877244 | 0.355272627 | -2.74927098340304 | 2.28E-05 |
| Zim1 | 7.67789346 | 1.130154596 | -2.76419040198842 | 4.24E-40 |
| Arc | 2.85677169 | 0.419498113 | -2.7676495137128 | 5.33E-14 |
| Vat1l | 7.77810247 | 1.141052083 | -2.76905359865483 | 2.13E-42 |
| Racgap1 | 7.87037908 | 1.151737271 | -2.77262146932372 | 3.03E-36 |
| Mcm5 | 3.82551763 | 0.559391773 | -2.77372402671303 | 1.89E-20 |
| Kcnn1 | 2.9009315 | 0.423303555 | -2.77675172579459 | 2.18E-18 |
| Tgfbi | 23.2968096 | 3.394193484 | -2.77899167976731 | 1.92E-91 |
| Myl9 | 38.9156633 | 5.628266347 | -2.78958844165379 | 1.45E-62 |
| Parpbp | 1.43304061 | 0.206328397 | -2.79606519253684 | 6.45E-09 |
| Ccnb1 | 5.15837747 | 0.738770712 | -2.80371876481378 | 6.93E-19 |
| Slfn9 | 4.8481673 | 0.693316005 | -2.80585451487352 | 8.92E-29 |
| Hopx | 5.33417461 | 0.760817032 | -2.80964360287488 | 2.95E-11 |
| Trp63 | 1.26078679 | 0.179826896 | -2.80964360287488 | 2.95E-11 |
| Proser3 | 1.83915657 | 0.260187495 | -2.82142076915887 | 5.42E-08 |
| Ddah2 | 45.4765105 | 6.416223635 | -2.82532522040897 | 1.39E-91 |
| Rtn4r | 3.24654197 | 0.456508263 | -2.83019098307714 | 2.43E-10 |
| Flrt1 | 1.30228275 | 0.182455316 | -2.83542763237602 | 1.13E-12 |
| Lama2 | 1.75404191 | 0.242237987 | -2.856186193289 | 8.07E-27 |
| Kif20a | 4.543427 | 0.627459702 | -2.856186193289 | 1.15E-25 |
| Wnt16 | 8.366108 | 1.155382407 | -2.856186193289 | 3.35E-22 |
| F8 | 1.34092008 | 0.185184733 | -2.856186193289 | 2.06E-16 |
| Chst5 | 1.6006817 | 0.221058523 | -2.856186193289 | 7.09E-06 |
| 6330403K07Rik | 16.5443247 | 2.275256608 | -2.8622352304561 | 6.86E-41 |
| Ckap2l | 2.50363923 | 0.340889578 | -2.87665029629642 | 3.52E-13 |
| Unc5c | 5.54570762 | 0.754446392 | -2.87788125520803 | 3.84E-79 |
| Adamts17 | 1.04582132 | 0.141896912 | -2.88172128520959 | 7.59E-11 |
| Fzd4 | 8.58531945 | 1.160782204 | -2.88677451103311 | 4.79E-49 |
| Eya1 | 3.46479072 | 0.467941997 | -2.8883665896127 | 4.10E-24 |
| Galnt16 | 2.4268353 | 0.327358349 | -2.89013351949161 | 9.29E-16 |
| Cml3 | 2.58651288 | 0.348897424 | -2.89013351949161 | 1.68E-08 |
| Cmbl | 3.82542498 | 0.513626612 | -2.89682817659245 | 2.55E-07 |
| Ramp1 | 1.56228615 | 0.209762771 | -2.89682817659245 | 2.55E-07 |
| E2f8 | 1.07891287 | 0.144861908 | -2.89682817659245 | 2.55E-07 |
| Dock10 | 1.41973548 | 0.189811822 | -2.90298040339037 | 3.58E-17 |
| Wscd2 | 0.74568518 | 0.099430089 | -2.90681226300115 | 3.93E-06 |
| 2810417H13Rik | 4.40249737 | 0.582397062 | -2.91824716096206 | 2.00E-17 |
| Prkcb | 2.56844496 | 0.339136674 | -2.92095644926552 | 5.26E-36 |
| Sh3rf2 | 2.54348648 | 0.335295968 | -2.92330038815262 | 5.37E-20 |
| Slc7a3 | 2.01457604 | 0.26557217 | -2.92330038815262 | 9.36E-09 |
| Clec3a | 1.66116577 | 0.218983742 | -2.92330038815262 | 9.36E-09 |
| Shank3 | 1.1480746 | 0.149981852 | -2.93635653361575 | 6.08E-14 |
| Robo2 | 7.53311289 | 0.983390392 | -2.93740998017347 | 3.99E-94 |
| Park2 | 1.7969897 | 0.233851741 | -2.94191606134543 | 3.48E-10 |
| Cdh3 | 1.83587865 | 0.238403157 | -2.94499545510649 | 8.88E-13 |
| Epha3 | 2.50268188 | 0.322585401 | -2.95572186609373 | 8.91E-17 |
| Prex2 | 0.30021315 | 0.03869624 | -2.95572186609373 | 2.17E-06 |
| Cacna1d | 2.45041068 | 0.315069908 | -2.95927967928874 | 3.99E-36 |
| Ace | 3.12886852 | 0.39909726 | -2.97082875294649 | 3.80E-26 |
| Cacnb2 | 5.55195709 | 0.704781641 | -2.97774817259592 | 1.74E-35 |
| Rpl22l1 | 32.0028478 | 4.035360777 | -2.98743072010035 | 3.00E-25 |
| Pianp | 2.28489124 | 0.288110626 | -2.98743072010035 | 2.87E-09 |
| Nckap1l | 0.53937384 | 0.068011698 | -2.98743072010035 | 3.42E-05 |
| Hvcn1 | 5.19391707 | 0.651587824 | -2.99479142800706 | 4.28E-24 |
| Hoxa13 | 4.57031454 | 0.572731005 | -2.99636384707336 | 1.06E-10 |
| Smad9 | 3.37201495 | 0.417412318 | -3.01406584710127 | 5.47E-30 |
| Tnik | 0.85509763 | 0.105208633 | -3.02283606248525 | 5.87E-11 |
| Mlip | 2.16899096 | 0.262100639 | -3.04883127123139 | 1.88E-05 |
| Sel1l3 | 0.58839648 | 0.071101769 | -3.04883127123139 | 1.88E-05 |
| Ticrr | 0.36893343 | 0.044581876 | -3.04883127123139 | 1.88E-05 |
| Spock2 | 3.87094249 | 0.464349944 | -3.05940051267822 | 1.22E-25 |
| C1qtnf2 | 18.7256817 | 2.207618922 | -3.08445517499266 | 6.12E-38 |
| Vit | 1.6674118 | 0.196575479 | -3.08445517499266 | 1.30E-08 |
| Cables1 | 3.27587059 | 0.383860223 | -3.09322539096294 | 4.26E-19 |
| Ranbp17 | 0.72979044 | 0.085515442 | -3.09322539096294 | 3.60E-07 |
| Nog | 5.23740221 | 0.612022719 | -3.09719428329594 | 1.11E-17 |
| Aspm | 1.02030074 | 0.119228428 | -3.09719428329594 | 1.11E-17 |
| Pag1 | 3.23022754 | 0.375763329 | -3.10373961063558 | 1.34E-44 |
| Cttnbp2 | 2.48057085 | 0.28848305 | -3.10411370477386 | 2.88E-25 |
| Tnmd | 14.6099957 | 1.694851526 | -3.10772495083333 | 7.85E-33 |
| Plk1 | 3.76595117 | 0.436874056 | -3.10772495083333 | 7.61E-15 |
| Nckap5 | 1.41091163 | 0.163081612 | -3.11296160322434 | 6.09E-18 |
| Satb1 | 1.32875122 | 0.152115307 | -3.12683377792649 | 4.17E-15 |
| Kcnq4 | 0.74861272 | 0.085140972 | -3.13629411173555 | 0.000305 |
| Ccdc158 | 0.51719339 | 0.058821265 | -3.13629411173555 | 0.000305 |
| Cml5 | 1.90721619 | 0.216910873 | -3.13629411173555 | 0.000305 |
| 0610040J01Rik | 1.00562308 | 0.114371188 | -3.13629411173555 | 0.000305 |
| Egr3 | 2.42374379 | 0.27245111 | -3.15316792746446 | 4.77E-17 |
| Lhfpl4 | 0.99823655 | 0.112210976 | -3.15316792746446 | 3.88E-09 |
| Rab3il1 | 18.4999971 | 2.074210227 | -3.15689100780304 | 5.40E-72 |
| Ldlrad3 | 11.7125512 | 1.312782329 | -3.15735572726338 | 1.02E-76 |
| Pard3b | 3.44035256 | 0.382282152 | -3.16984667099887 | 3.94E-49 |
| Dpysl4 | 1.46708938 | 0.162087246 | -3.17811428556473 | 1.07E-07 |
| Ackr4 | 8.81171862 | 0.963396942 | -3.19322117616803 | 2.28E-32 |
| Dpp4 | 1.30188865 | 0.142095563 | -3.19567265572316 | 8.70E-13 |
| Reps2 | 2.44572137 | 0.264248166 | -3.21029468984115 | 6.79E-33 |
| Myh11 | 0.45034689 | 0.048373218 | -3.21875627416607 | 3.02E-06 |
| Sh3tc2 | 0.43857571 | 0.047108837 | -3.21875627416607 | 0.000165 |
| Aass | 0.53799884 | 0.057788198 | -3.21875627416607 | 0.000165 |
| Myl1 | 2.00315262 | 0.215165112 | -3.21875627416607 | 0.000165 |
| Kctd14 | 0.84945977 | 0.091243226 | -3.21875627416607 | 0.000165 |
| Smoc2 | 12.7336313 | 1.359369414 | -3.22763441299326 | 5.26E-62 |
| Fam101a | 4.89403233 | 0.518482718 | -3.2386558284325 | 5.31E-15 |
| Phactr1 | 2.86316642 | 0.303052316 | -3.23997275350937 | 5.29E-27 |
| Tnn | 1.04734372 | 0.110453153 | -3.24522848577598 | 1.26E-11 |
| Meox1 | 18.0857303 | 1.888833951 | -3.25928408097827 | 4.17E-69 |
| Tenm3 | 5.19941393 | 0.535756817 | -3.27869881421534 | 1.30E-98 |
| Mpped2 | 3.90613925 | 0.401716982 | -3.28149202772319 | 3.69E-19 |
| Plin4 | 7.26564861 | 0.74326367 | -3.28914560004278 | 6.92E-73 |
| Tnnt3 | 5.83820067 | 0.594094781 | -3.29675878094407 | 3.70E-12 |
| Zkscan16 | 0.75223774 | 0.076547645 | -3.29675878094407 | 8.88E-05 |
| Ror1 | 5.10869696 | 0.515980992 | -3.30756553131697 | 2.15E-52 |
| Cdca3 | 4.31174413 | 0.431197825 | -3.32184975867345 | 2.00E-12 |
| Unc5a | 0.80723141 | 0.080727524 | -3.32184975867345 | 8.80E-07 |
| Ifit3b | 9.34185377 | 0.903094898 | -3.37075936910348 | 2.47E-34 |
| Slc16a4 | 6.57841266 | 0.635947753 | -3.37075936910348 | 1.39E-26 |
| Ucp2 | 7.01894283 | 0.678534648 | -3.37075936910348 | 9.24E-21 |
| Pax1 | 3.35461832 | 0.324297379 | -3.37075936910348 | 7.22E-17 |
| P2ry1 | 1.41855013 | 0.137133958 | -3.37075936910348 | 5.31E-11 |
| Lfng | 1.45295804 | 0.140460237 | -3.37075936910348 | 4.73E-07 |
| Zfp711 | 0.7664102 | 0.074090342 | -3.37075936910348 | 4.73E-07 |
| Cep55 | 0.80129329 | 0.077462557 | -3.37075936910348 | 4.76E-05 |
| Ifi47 | 1.14216354 | 0.110415137 | -3.37075936910348 | 4.76E-05 |
| Aard | 1.64122462 | 0.158660327 | -3.37075936910348 | 4.76E-05 |
| Asxl3 | 0.18914002 | 0.018284528 | -3.37075936910348 | 4.76E-05 |
| Scrn1 | 30.8760807 | 2.948742807 | -3.38831773555385 | 1.07E-97 |
| Cd36 | 2.20244427 | 0.209915711 | -3.3912234676125 | 3.47E-15 |
| Pknox2 | 6.35667098 | 0.602462313 | -3.3993285099583 | 3.29E-41 |
| Enpp3 | 2.00410142 | 0.189941493 | -3.3993285099583 | 2.86E-11 |
| Fmo1 | 8.27498959 | 0.78156923 | -3.40431191547103 | 2.09E-35 |
| Ildr2 | 4.12475908 | 0.388391261 | -3.40872720656768 | 1.35E-61 |
| Nkain4 | 8.38372085 | 0.787957289 | -3.4114013386772 | 1.87E-15 |
| Gpd1 | 4.92520605 | 0.460770099 | -3.41806507045058 | 1.02E-25 |
| Nkd1 | 7.46175124 | 0.69407457 | -3.42635168222182 | 1.53E-58 |
| Adamts12 | 3.15850176 | 0.29161534 | -3.43710186129859 | 1.76E-36 |
| Cdk15 | 3.14555087 | 0.289606122 | -3.44114868878688 | 1.42E-09 |
| Lgi4 | 1.34900657 | 0.124201 | -3.44114868878688 | 1.42E-09 |
| Tprg | 2.14297908 | 0.19730085 | -3.44114868878688 | 2.54E-05 |
| Cdh6 | 0.88259473 | 0.081259165 | -3.44114868878688 | 2.54E-05 |
| Crispld2 | 2.29904752 | 0.204370875 | -3.49177476913212 | 9.33E-19 |
| Arfgef3 | 0.29620349 | 0.026031417 | -3.50826287792979 | 1.35E-05 |
| Pcdhb21 | 0.89734803 | 0.078862139 | -3.50826287792979 | 1.35E-05 |
| Tmem56 | 0.99560241 | 0.085934636 | -3.53425809840163 | 1.26E-12 |
| Cybrd1 | 1.9116107 | 0.164265838 | -3.54068435562217 | 1.43E-19 |
| Sgol1 | 1.07277608 | 0.088891987 | -3.59315178049085 | 2.05E-08 |
| Cav3 | 3.34628251 | 0.277278463 | -3.59315178049085 | 2.05E-08 |
| Kif11 | 2.68569601 | 0.22190716 | -3.59726788801792 | 2.29E-25 |
| Adcy1 | 0.424137 | 0.034895435 | -3.6034201120894 | 6.14E-11 |
| Fap | 5.31819899 | 0.435025025 | -3.6117674631805 | 4.00E-28 |
| Syt9 | 0.69571408 | 0.056046677 | -3.63379376001365 | 3.80E-06 |
| Fgf14 | 2.50470017 | 0.199014381 | -3.65369332317208 | 1.68E-16 |
| Eepd1 | 8.15258219 | 0.640102121 | -3.67088309208686 | 1.91E-42 |
| Plxdc1 | 3.76978842 | 0.294491045 | -3.6781878793721 | 4.87E-22 |
| Myh14 | 1.05270137 | 0.082069885 | -3.68109948553702 | 2.83E-14 |
| Cilp | 1.35842656 | 0.10299741 | -3.72125661245671 | 4.83E-12 |
| Rab11fip4 | 6.29593593 | 0.47603143 | -3.72529212931617 | 3.83E-39 |
| 9930012K11Rik | 2.89635651 | 0.215381794 | -3.74927098340304 | 2.55E-12 |
| Adh7 | 1.38339683 | 0.102873555 | -3.74927098340304 | 1.06E-06 |
| Popdc3 | 1.55379904 | 0.115545176 | -3.74927098340304 | 1.06E-06 |
| Abcb11 | 0.58755506 | 0.043692364 | -3.74927098340304 | 1.06E-06 |
| Tmem151b | 0.29644218 | 0.022044333 | -3.74927098340304 | 0.00078 |
| Cldn20 | 2.17885001 | 0.162025849 | -3.74927098340304 | 0.00078 |
| Adamts16 | 0.28911158 | 0.021499208 | -3.74927098340304 | 0.00078 |
| Lyl1 | 0.79846808 | 0.059376491 | -3.74927098340304 | 0.00078 |
| Iqgap3 | 1.28626208 | 0.094201075 | -3.77129728376359 | 2.21E-15 |
| Ccnb2 | 5.8144722 | 0.41636753 | -3.80371877488722 | 1.03E-18 |
| Slc4a10 | 0.53989526 | 0.038661266 | -3.80371877488722 | 5.55E-07 |
| Fjx1 | 7.61157644 | 0.528737012 | -3.84757306808319 | 1.62E-37 |
| Bmp3 | 1.9303952 | 0.13329643 | -3.856186193289 | 1.96E-13 |
| Dscc1 | 1.18308598 | 0.081693706 | -3.856186193289 | 0.000408 |
| Scn7a | 0.21119045 | 0.014582989 | -3.856186193289 | 0.000408 |
| Kif19a | 0.45110968 | 0.031149741 | -3.856186193289 | 0.000408 |
| Ankle1 | 0.55867949 | 0.038577583 | -3.856186193289 | 0.000408 |
| Epha1 | 0.4731621 | 0.03267249 | -3.856186193289 | 0.000408 |
| Lrrc4b | 0.56956953 | 0.039329555 | -3.856186193289 | 0.000408 |
| Syndig1 | 0.75360562 | 0.052037499 | -3.856186193289 | 0.000408 |
| Abcc6 | 0.3123557 | 0.021568588 | -3.856186193289 | 0.000408 |
| Ntng1 | 0.28963148 | 0.01999945 | -3.856186193289 | 0.000408 |
| Aoc3 | 3.54804686 | 0.243259919 | -3.86645452844435 | 5.28E-32 |
| Tmem44 | 3.31255705 | 0.226935521 | -3.86759094478817 | 5.77E-29 |
| Cthrc1 | 18.8165556 | 1.286184578 | -3.8708329573145 | 2.02E-44 |
| Me3 | 2.53131668 | 0.171295063 | -3.88533253127351 | 3.69E-23 |
| Ccdc3 | 13.5672098 | 0.885107349 | -3.93812780194108 | 4.03E-73 |
| Akr1c14 | 2.71456538 | 0.174948156 | -3.95572185490101 | 1.48E-14 |
| Neurl1b | 0.53628897 | 0.034562722 | -3.95572185490101 | 7.96E-08 |
| Dock2 | 0.25861566 | 0.016667248 | -3.95572185490101 | 0.000212 |
| Tnnc2 | 2.3978007 | 0.154533325 | -3.95572185490101 | 0.000212 |
| Jph3 | 0.4251289 | 0.027398683 | -3.95572185490101 | 0.000212 |
| Mia | 41.5551126 | 2.640421249 | -3.97618596833365 | 1.27E-48 |
| B3galt1 | 1.29217367 | 0.081912724 | -3.97956860431719 | 7.72E-15 |
| Fign | 0.68735162 | 0.043572195 | -3.97956860431719 | 7.72E-15 |
| Hpgd | 3.92538155 | 0.24883551 | -3.97956860431719 | 7.72E-15 |
| Sfxn4 | 1.86321963 | 0.117470224 | -3.98743073154179 | 1.74E-11 |
| Cacna2d3 | 3.2332347 | 0.202587124 | -3.99636384707336 | 2.09E-25 |
| Clec14a | 3.85056822 | 0.23559608 | -4.03068391168682 | 8.90E-37 |
| Slc9a9 | 12.1325603 | 0.734962616 | -4.04506933887957 | 2.51E-87 |
| Pla2g5 | 2.48930608 | 0.150403742 | -4.04883127123139 | 4.74E-12 |
| Hunk | 1.06002992 | 0.064046952 | -4.04883127123139 | 4.74E-12 |
| Doc2b | 0.83387356 | 0.050382596 | -4.04883127123139 | 2.16E-08 |
| Myh1 | 0.58810322 | 0.035533165 | -4.04883127123139 | 2.16E-08 |
| Rnf112 | 1.14260596 | 0.069036191 | -4.04883127123139 | 2.16E-08 |
| Lrrn1 | 0.47121848 | 0.028470996 | -4.04883127123139 | 0.00011 |
| Hs3st3a1 | 0.45001186 | 0.027189692 | -4.04883127123139 | 0.00011 |
| Gpr174 | 0.33964625 | 0.020521409 | -4.04883127123139 | 0.00011 |
| Nptx1 | 5.04524374 | 0.303568491 | -4.05483000767422 | 1.35E-55 |
| Efhd1 | 3.9834931 | 0.236979636 | -4.07119906859004 | 5.71E-16 |
| Thsd4 | 2.0498888 | 0.121574648 | -4.07563131684538 | 3.44E-38 |
| Igfbp3 | 17.0008001 | 1.008013276 | -4.07601610029808 | 5.32E-86 |
| Klf15 | 4.72563942 | 0.279695886 | -4.07857861114327 | 1.72E-23 |
| Cacnb4 | 0.91635041 | 0.052886776 | -4.11492046765861 | 1.55E-16 |
| Dkk2 | 2.00038924 | 0.115451617 | -4.11492046765861 | 1.55E-16 |
| Lrrn3 | 3.73966084 | 0.212658927 | -4.13629409905047 | 1.61E-28 |
| Tmem100 | 2.13210336 | 0.121243833 | -4.13629409905047 | 5.82E-09 |
| Pex5l | 0.36858392 | 0.020959832 | -4.13629409905047 | 5.70E-05 |
| Tacr1 | 0.37415741 | 0.021276773 | -4.13629409905047 | 5.70E-05 |
| Susd2 | 7.57847441 | 0.427091179 | -4.1492914531254 | 2.23E-52 |
| Shcbp1 | 2.69801315 | 0.150474288 | -4.16430848268188 | 3.46E-13 |
| Fras1 | 1.71707222 | 0.094467368 | -4.18399085728456 | 5.84E-58 |
| Lcp2 | 0.59472333 | 0.03194058 | -4.21875626073481 | 2.94E-05 |
| Olfml2a | 11.7093099 | 0.621469011 | -4.23582978528647 | 1.59E-60 |
| Slurp1 | 7.79597323 | 0.407379278 | -4.25828464506833 | 8.06E-10 |
| Gm4951 | 1.58742617 | 0.080768173 | -4.29675876676663 | 4.16E-10 |
| Igfals | 1.99123851 | 0.101314127 | -4.29675876676663 | 4.16E-10 |
| C8g | 2.01317265 | 0.102430135 | -4.29675876676663 | 1.51E-05 |
| Chn2 | 0.66722293 | 0.033948273 | -4.29675876676663 | 1.51E-05 |
| Fancb | 0.71101226 | 0.036176272 | -4.29675876676663 | 1.51E-05 |
| Tspan18 | 2.83107965 | 0.142544736 | -4.31186567228963 | 7.17E-24 |
| Arsi | 12.3980128 | 0.621004998 | -4.31936022032774 | 9.71E-47 |
| Upb1 | 0.72727508 | 0.035153537 | -4.37075935417986 | 7.78E-06 |
| Mboat1 | 0.76791766 | 0.037118036 | -4.37075935417986 | 7.78E-06 |
| Tspan8 | 1.45359447 | 0.070260881 | -4.37075935417986 | 7.78E-06 |
| Angptl1 | 12.2236011 | 0.585956496 | -4.38273200405393 | 8.51E-59 |
| Wfdc3 | 2.83291381 | 0.130411049 | -4.44114870445669 | 3.99E-06 |
| Dlx5 | 3.37347318 | 0.151683774 | -4.47509600951751 | 1.50E-11 |
| Dmp1 | 0.88174198 | 0.038745312 | -4.50826287792979 | 2.04E-06 |
| Sncaip | 3.9464232 | 0.172109003 | -4.51915121645796 | 1.58E-33 |
| Fat3 | 0.55105618 | 0.02316159 | -4.57239320938006 | 9.97E-24 |
| Cyp4f14 | 2.3045529 | 0.09686328 | -4.57239320938006 | 2.02E-12 |
| Rbp1 | 9.01117905 | 0.366362217 | -4.62037325386877 | 7.62E-54 |
| Aldh1a7 | 15.6799094 | 0.622934334 | -4.65369334132915 | 2.86E-73 |
| Col28a1 | 2.56279358 | 0.101122516 | -4.6635410996768 | 1.79E-25 |
| Mamstr | 2.48752107 | 0.098152419 | -4.6635410996768 | 2.69E-13 |
| Cenpf | 1.48194231 | 0.057689477 | -4.6830372909724 | 3.53E-38 |
| Amigo2 | 1.7208858 | 0.066544531 | -4.69268746399084 | 1.38E-13 |
| Fzd9 | 12.1103709 | 0.456129642 | -4.73065529379425 | 8.47E-59 |
| Edar | 2.31457269 | 0.087176952 | -4.73065529379425 | 2.03E-20 |
| Shmt1 | 2.78555159 | 0.103571003 | -4.74927098340304 | 3.58E-14 |
| Abca9 | 4.64146072 | 0.170607946 | -4.76582217369509 | 4.97E-67 |
| Plekhg4 | 2.09715973 | 0.076988525 | -4.7676495137128 | 5.29E-21 |
| B4galnt2 | 2.89676546 | 0.104686305 | -4.79029824643982 | 4.20E-28 |
| Smarca1 | 4.39200611 | 0.158229929 | -4.79478563643723 | 1.10E-41 |
| Dio3 | 1.5988761 | 0.05724682 | -4.80371877488722 | 7.04E-08 |
| Aox3 | 0.68502306 | 0.024526849 | -4.80371877488722 | 7.04E-08 |
| Pappa2 | 0.34789756 | 0.012456268 | -4.80371877488722 | 7.04E-08 |
| Adgrl3 | 1.6368194 | 0.05519807 | -4.89013353018687 | 4.67E-23 |
| Plxnc1 | 1.3662426 | 0.045543893 | -4.90681227382078 | 2.37E-23 |
| C1qa | 3.06705746 | 0.098832773 | -4.95572185490101 | 9.20E-09 |
| Adra1b | 4.9727298 | 0.159179906 | -4.96530791328133 | 6.84E-40 |
| Fam107a | 1.09070442 | 0.03401306 | -5.00302757117173 | 4.66E-09 |
| Hmcn1 | 2.08987739 | 0.06422997 | -5.02402787426137 | 3.01E-90 |
| Scube1 | 2.20344543 | 0.067408636 | -5.03068393526614 | 5.91E-42 |
| Col6a6 | 1.49610873 | 0.045197405 | -5.0488312473536 | 5.28E-26 |
| Adam12 | 4.67036834 | 0.139349831 | -5.06675317266226 | 5.82E-85 |
| Tspan13 | 9.54897425 | 0.278047479 | -5.10194259515511 | 2.56E-44 |
| Megf6 | 3.76450526 | 0.108865536 | -5.1118410683308 | 5.25E-62 |
| Slc38a4 | 7.53961558 | 0.21757286 | -5.11492046765861 | 7.63E-71 |
| Atp1a2 | 1.20797505 | 0.034346254 | -5.13629412442063 | 6.89E-19 |
| Cyb5r2 | 1.39669873 | 0.038577583 | -5.17811429862289 | 3.04E-10 |
| Mdk | 10.989957 | 0.297874821 | -5.20533577233246 | 2.92E-29 |
| Prokr1 | 3.9000126 | 0.105460612 | -5.20870261659086 | 1.01E-38 |
| Tll1 | 0.80808998 | 0.021699891 | -5.21875626073481 | 1.53E-10 |
| Angpt4 | 2.60278915 | 0.069893504 | -5.21875626073481 | 1.53E-10 |
| Kcnma1 | 1.32156472 | 0.034529241 | -5.25828461745963 | 1.14E-20 |
| Col15a1 | 1.55976507 | 0.040209461 | -5.27764998336652 | 5.76E-21 |
| Trpm3 | 1.26336807 | 0.031722652 | -5.31561783281936 | 1.46E-21 |
| Cilp2 | 6.54580117 | 0.159528186 | -5.35868653730036 | 1.92E-64 |
| Fam64a | 3.0936972 | 0.072944789 | -5.40638326988003 | 4.96E-12 |
| Trim63 | 2.40475085 | 0.056700456 | -5.40638326988003 | 4.96E-12 |
| C1qc | 4.51290055 | 0.101458312 | -5.47509600951751 | 1.25E-12 |
| Pgm5 | 4.19965134 | 0.093792686 | -5.48465031687034 | 1.61E-82 |
| Ggt5 | 6.95209576 | 0.155093634 | -5.48623655170163 | 5.27E-71 |
| Abcc9 | 0.6456906 | 0.014186397 | -5.50826287792979 | 6.30E-13 |
| Nos1 | 0.51344345 | 0.011030125 | -5.54068435562217 | 3.16E-13 |
| Myoc | 9.73230416 | 0.205648193 | -5.56453112779687 | 1.21E-50 |
| Adh1 | 8.20932186 | 0.160325428 | -5.67818787937211 | 3.89E-28 |
| Scg2 | 4.40693576 | 0.086066045 | -5.67818787937211 | 3.89E-28 |
| Col24a1 | 4.63429216 | 0.08990085 | -5.68787040963783 | 1.12E-82 |
| Stk32b | 1.63998415 | 0.031086355 | -5.72125657440147 | 5.02E-15 |
| Slc27a6 | 4.42814783 | 0.082322602 | -5.74927098340304 | 1.23E-29 |
| Kcns1 | 4.28279742 | 0.078862139 | -5.76307679934405 | 6.14E-30 |
| Galnt9 | 6.04556526 | 0.110968932 | -5.7676495137128 | 1.69E-44 |
| Ptprq | 0.84633056 | 0.015151185 | -5.80371873459345 | 6.29E-16 |
| Wdr5b | 3.47038554 | 0.059908717 | -5.856186193289 | 1.57E-16 |
| St6galnac2 | 6.25732021 | 0.10520124 | -5.89432132142959 | 6.03E-33 |
| Acox2 | 2.53653082 | 0.041561236 | -5.9314743534252 | 1.96E-17 |
| Wif1 | 3.05903186 | 0.047697172 | -6.00302761743496 | 2.44E-18 |
| Hsd11b2 | 7.83722478 | 0.115670157 | -6.08225427500739 | 9.02E-38 |
| Vstm4 | 8.14617021 | 0.116956319 | -6.12208026619584 | 9.32E-58 |
| Plin1 | 3.92591892 | 0.055812662 | -6.13629412442063 | 3.77E-20 |
| Ramp2 | 10.2753459 | 0.122634244 | -6.38868131590216 | 4.36E-24 |
| Phex | 1.76565904 | 0.017068964 | -6.69268738937274 | 7.37E-30 |
| Adipoq | 9.51750417 | 0.086799562 | -6.77675172579459 | 1.10E-31 |
| col1a2 | 3.74559518 | 0.019895267 | -7.55662582785781 | 6.75E-55 |
| lrs1 | 16.2010345 | 0.064187912 | -7.97956851328311 | 6.79E-74 |
| Thbs1 | 8.35111041 | 0.032553139 | -8.00302761743496 | 4.03E-75 |
| Nos2 | 17.5555019 | 0.060828817 | -8.1729526651405 | 1.24E-84 |
